# METTL3 and STAT3 form a positive feedback loop to promote cell metastasis in hepatocellular carcinoma

**DOI:** 10.1186/s12964-023-01148-7

**Published:** 2023-05-25

**Authors:** Bowen Liu, Jinling Cao, Biting Wu, Kaixuan Hao, Xiangyun Wang, Xin Chen, Zhifa Shen

**Affiliations:** 1 Laboratory of Infection and Immunology, School of Medical Technology, Xinxiang Medical University, Xinxiang, Henan 453003 People’s Republic of China; 2 Key Laboratory of Laboratory Medicine, Ministry of Education of China, Zhejiang Provincial Key Laboratory of Medical Genetics, School of Laboratory Medicine and Life Sciences, Wenzhou Medical University, Wenzhou, 325035 China

**Keywords:** Hepatocellular carcinoma, m6A methylation modification, METTL3, STAT3, Metastasis

## Abstract

**Background:**

It is well-established that most Hepatocellular carcinoma (HCC) patients die of metastasis, yet the potential mechanisms orchestrating metastasis remain poorly understood. Current evidence suggests that the dysregulation of METTL3-mediated m6A methylation modification is closely associated with cancer progression. STAT3 is an oncogenic transcription factor that reportedly plays a central role in the occurrence and development of HCC. However, the relationship between METTL3 and STAT3 in HCC metastasis remains unclear.

**Methods:**

The relationship between METTL3 expression and the survival of HCC patients was assessed by online tools GEPIA and Kaplan–Meier Plotter. Western blotting, Tissue microarray (TMA), and immunohistochemistry (IHC) staining were used to evaluate the expression levels of METTL3 and STAT3 in HCC cell lines and metastatic and non-metastatic tissues. Methylated RNA immunoprecipitation (MeRIP), MeRIP sequencing (MeRIP-seq), qRT-PCR, RNA immunoprecipitation (RIP), Western blotting and luciferase reporter gene assay were utilized to clarify the mechanism of METTL3 regulating STAT3 expression. Immunofluorescence staining, Western blotting, qRT-PCR, Co-immunoprecipitation (Co-IP), IHC staining, TMA and Chromatin immunoprecipitation (ChIP) assay were performed to explore the mechanism of STAT3 modulating METTL3 localization. Cell viability, wound healing and transwell assay, and orthotopic xenograft model were used to evaluate the role of METTL3-STAT3 feedback loop in the promotion of HCC metastasis in vitro and in vivo.

**Results:**

METTL3 and STAT3 are both abundantly expressed in high-metastatic HCC cells and tissues. Moreover, a positive correlation was found between the expression of STAT3 and METTL3 in HCC tissues. Mechanistically, METTL3 could induce the m6A modification of STAT3 mRNA, and then promote the translation of m6A-contained STAT3 mRNA by interacting with the translation initiation machinery. In contrast, STAT3 promoted nuclear localization of METTL3 via transcriptionally upregulating WTAP, a vital member of the methyltransferase complex, and facilitated the methyltransferase function of METTL3. METTL3 and STAT3 form a positive feedback loop to accelerate HCC metastasis in vitro and in vivo.

**Conclusions:**

Our findings reveal a novel mechanism of HCC metastasis and uncover the METTL3-STAT3 feedback signaling as a potential target for the anti-metastatic treatment of HCC.

Video Abstract

**Supplementary Information:**

The online version contains supplementary material available at 10.1186/s12964-023-01148-7.

## Introduction

Hepatocellular carcinoma (HCC) is widely acknowledged as a common malignant tumor and a severe threat to human health [1]. Notwithstanding that significant progress has been made in clinical treatments for HCC patients in recent years, the mortality rate of HCC remains very high [2]. Frequent metastasis and recurrence have been reported to contribute to the high mortality of primary HCC [3]; nonetheless, the underlying mechanism remains to be investigated.

N6-methyladenosine (m6A) is a dynamic and reversible chemical modification in eukaryotes, which affects the fate of many mRNAs and non-coding RNAs by modulating RNA processing, transport, translation, and degradation [4, 5]. The m6A modification is catalyzed by the methyltransferase complex (MTC) composed of methyltransferase-like 3 (METTL3), methyltransferase-like 14 (METTL14), and the co-enzyme factors Wilms tumor 1 associated protein (WTAP), VIRMA (KIAA1429), and RBM15 [6, 7]. Members of the YT521-B homology (YTH) domain family and insulin-like growth factor 2 mRNA-binding protein family, such as YTHDF1/2/3 and IGF2BP1/2/3, can reportedly recognize m6A and modulate the fate of mRNA [8–10]. Recent studies have indicated that METTL3-mediated disorders of m6A modification are closely related to the occurrence and development of cancers [11, 12]. In gastric cancer, METTL3-mediated m6A modification enhances the stability of ZMYM1 mRNA and increases the expression of ZMYM1, thus facilitating epithelial-mesenchymal transition (EMT) and metastasis [13]. Moreover, METTL3 promotes the progression of intrahepatic cholangiocarcinoma by YTHDF2-mediated IFIT2 mRNA degradation [14]. Chen et al. reported that METTL3 contributes to the progression of HCC via the m6A-YTHDF2-SOCS2 axis [15]. Current evidence suggests that METTL3 is highly expressed in clinical HCC tissues, and its expression level is closely related to the poor prognosis of HCC patients [15]. However, the role of METTL3 in orchestrating cell metastasis in HCC remains largely understudied.

It is widely acknowledged that signal transducer and activator of transcription (STAT) family proteins can be activated by different cytokine receptors and participate in cell signal transmission and gene expression regulation [16]. Among these family members, STAT3 is an important oncogene that has been widely studied in recent years. A growing number of studies substantiate that STAT3 can affect the survival, proliferation, angiogenesis, and metastasis of cancer cells via regulating the expression of different genes, thereby promoting the malignant development of cancer [17–19]. One study revealed that high expression of STAT3 in HCC has been closely associated with poor prognosis [20]. However, whether m6A methylation modification is involved in regulating STAT3 expression in HCC is still unexplored.

In the present study, we discovered a positive correlation between STAT3 expression and METTL3 in HCC. METTL3 induced m6A modification and enhanced the translation of STAT3 mRNA, upregulating STAT3 protein levels. Moreover, STAT3 promoted nuclear localization of METTL3 by upregulating WTAP expression, potentiating the methyltransferase function of METTL3. Overall, METTL3 and STAT3 formed a positive feedback loop to accelerate HCC metastasis in vitro and in vivo. These findings provide a foothold for further studies on identifying novel therapeutic targets for HCC.

## Materials and methods

### Cell lines, transfection and treatments

The MHCC97H, MHCC97L, HCCLM3, HepG2, L-O2, SMCC-7721 and HEK293T cell lines were purchased from American Type Culture Collection (ATCC). L-O2 cells were cultured in RPMI Medium 1640 (Gibco, USA) supplemented with 10% FBS (Gibco). The other cell lines were cultured in high-glucose Dulbecco’s Modified Eagle’s Medium (DMEM; Gibco) with 10% FBS. The HCCLM3-KD-STAT3 (stably knockdown STAT3) and HCCLM3-control cell lines were gifts from Wenzhou medical university. The MHCC97H-control and MHCC97H-KD-STAT3 (stably knockdown STAT3) cell lines were stably transfected with pSilencer 4.1 CMV vector containing the nonsense RNAi fragment or STAT3 RNAi fragment. The related plasmid and RNA interference lentivirus were packed and purchased from GeneChem (Shanghai, China) to generate the stable cell lines used in Figs. 7 and 8. All cell lines tested negative for mycoplasma contamination. Streptomycin and penicillin (Sigma-Aldrich, USA) were added to the medium to inhibit bacteria. Lipofectamine 3000 reagent (Invitrogen, USA) was used for cell transfection according to the manufacturer’s recommendation. To analyze the stability of RNA, the indicated cells were treated with actinomycin D (Santa Cruz, USA) at 2 mg/ml for the corresponding time points. To evaluate the stability of protein, the indicated cells were treated with cycloheximide (CHX, MedChem Express, USA) at 100 μg/ml for the corresponding time points. STM2457 used in the study was purchased from MedChemExpress (#2,499,663–01-1).

### Immunohistochemistry (IHC) staining

The association of METTL3 and STAT3 was assessed by IHC staining in HCC tissue microarray (LV1221, aobosi Biological Technology Co., Ltd, China). The association between WTAP and STAT3 was assessed in HCC tissue microarray (DLV1505, aobosi Biological Technology Co., Ltd, China). The clinical characteristics of patients are presented in supplementary tables S1 and S2, respectively. The slides were incubated in a 65℃ dry oven for 1–2 h to remove paraffin. After dewaxing, antigen retrieval was performed by microwaving the samples in sodium citrate buffer (pH 8.0) for 20 min. The slides were incubated in a peroxidase blocker for 10 min, followed by closure in 5% BSA solution for 30 min. The slides were incubated with the corresponding primary antibodies overnight at 4 °C. After being washed with PBS twice, incubation with secondary antibody was performed at 37 °C for 1 h. 1 × DAB chromogenic solution (ZSGB-BIO, China) was added, and the slides were observed under a microscope. After staining, the reaction was terminated by putting the slides into the water. Finally, the slides were colored with hematoxylin (Solarbio, China) and sealed with neutral gum (Solarbio). After that, the slides were scanned for positive signals. In the analysis of tissue microarray, the staining levels of METTL3, STAT3 and WTAP in each case were quantitatively analyzed by Aipathwell software (Servicebio, China) and the corresponding H-scores were calculated (H-score = ∑ (pi × i) = (percentage of weak intensity × 1) + (percentage of moderate intensity × 2) + (percentage of strong intensity × 3) [21, 22]. Based on the H-scores, the scatter plots with linear regression line were generated.

## Patient samples

**Fig. 1:**
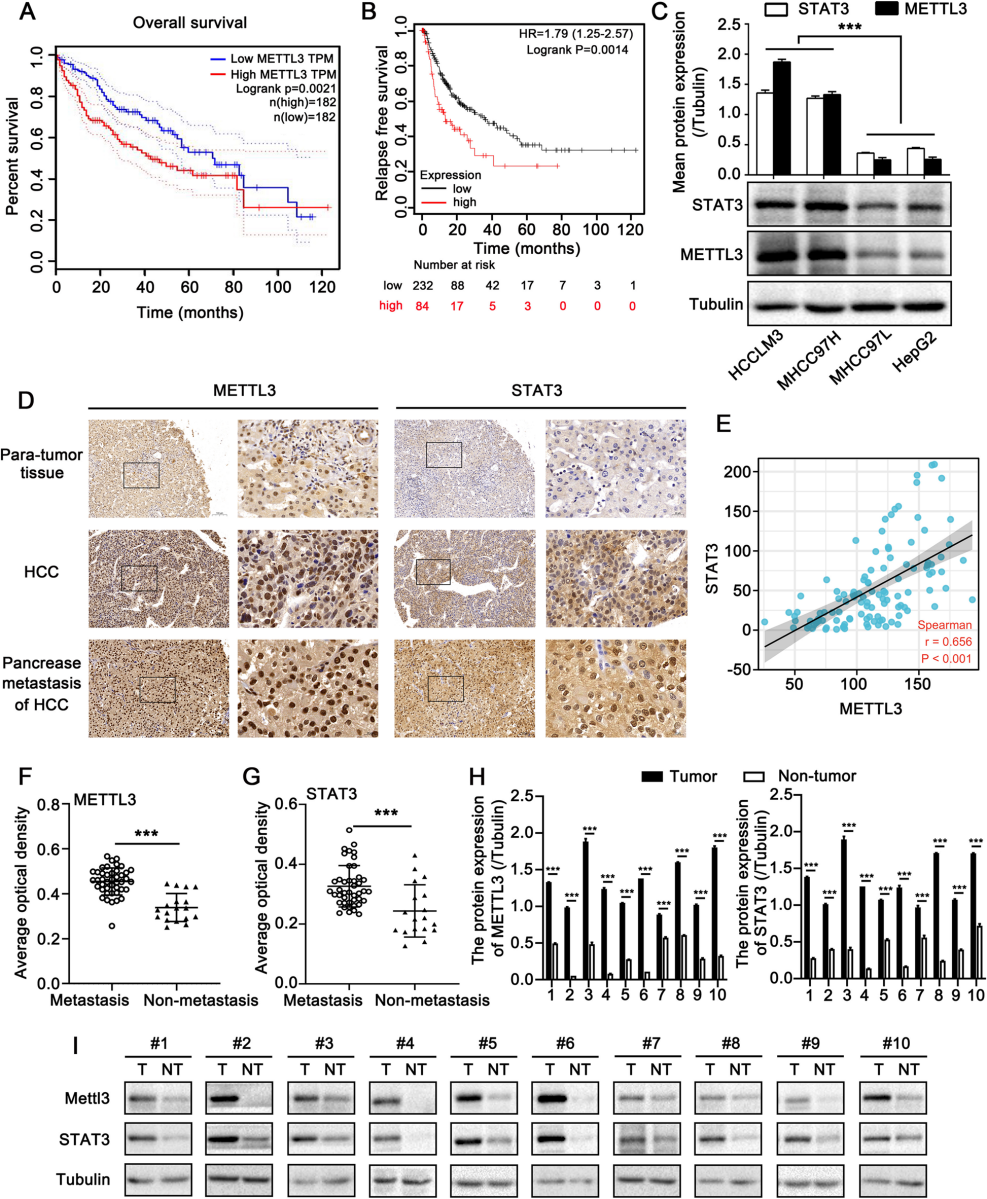
The expression of STAT3 is positively related to METTL3 in HCC. **A** The relationship between METTL3 and the overall survival of HCC patients was assessed by the GEPIA online tool. **B** The relationship between METTL3 and relapse-free survival of HCC patients was assessed by the Kaplan–Meier Plotter online tool. **C** Western blotting analysis of STAT3 and METTL3 in different HCC cell lines (lower panel). The upper panel is the quantification of the intensity relative to tubulin. **D** IHC staining of METTL3 and STAT3 in para-tumor tissues, HCC tissues and metastatic tissues from HCC tissue microarray. The right image is an enlarged view of the black box in the left image. Scale bar, left image:100 μm; right image, 20 μm. **E** The association between the expression levels of METTL3 and STAT3 in the tissue microarray was statistically analyzed by Spearman correlation analysis, *r* = 0.656, *p* < 0.001. **F**, **G** Statistics of average optical density of METTL3 (F) and STAT3 (G) in metastasis (*n* = 43) and non-metastasis (*n* = 18) tissues. **H**, **I** Western blotting analysis of STAT3 and METTL3 in 10 paired HCC tissues (T) and non-tumor tissues (NT). (H) shows the quantification of intensity of METTL3 and STAT3 relative to tubulin. All experiments were repeated at least three times. Error bars represent mean ± SD. *P < 0.05, **P < 0.01, ***P < 0.001 by 2-tailed Student’s *t-*test

**Fig. 2:**
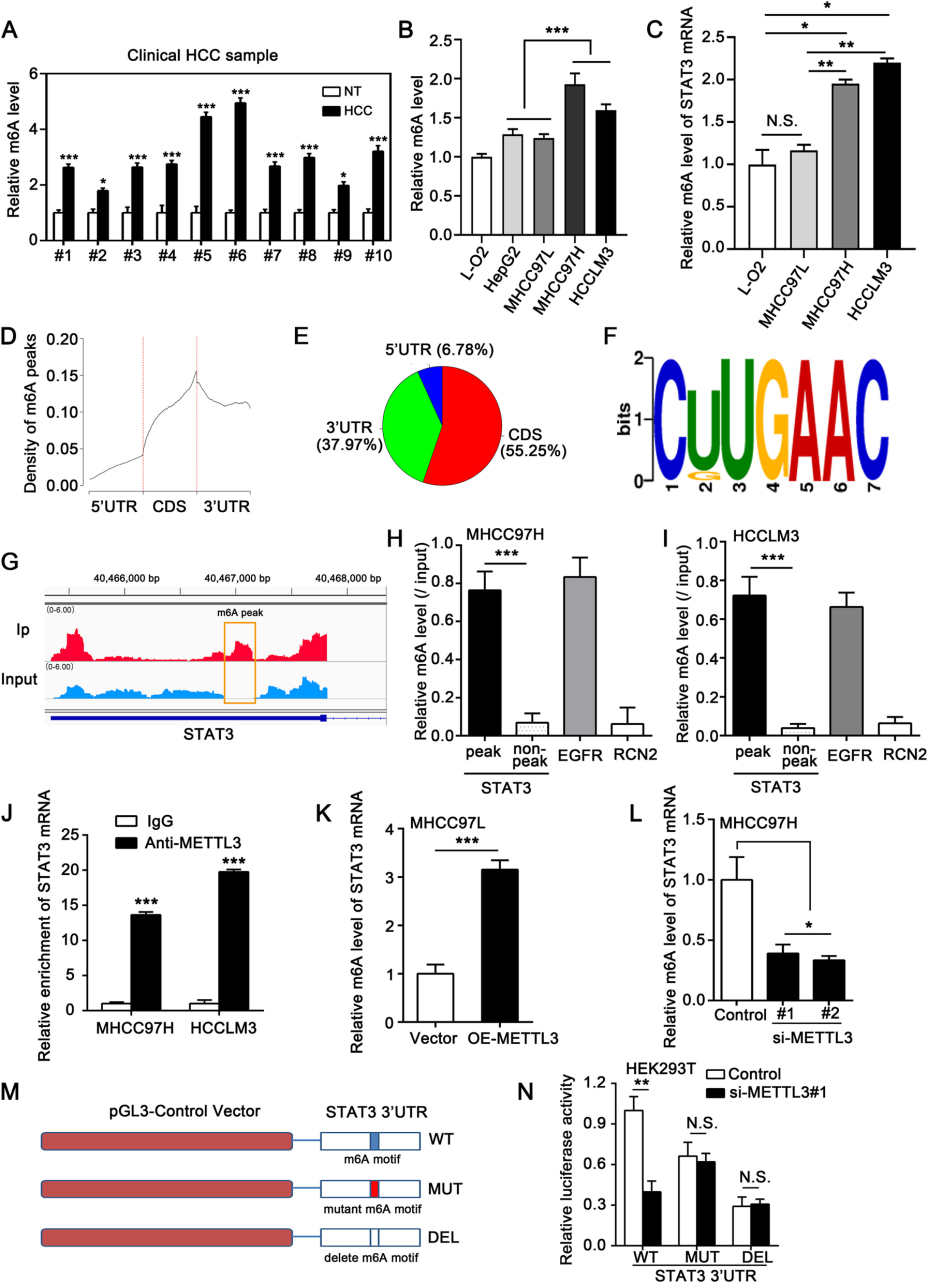
METTL3 mediates m6A modification of STAT3 mRNA in HCC cells. **A** The m6A level of total RNA in paired HCC samples (*n* = 10) was determined by the m6A RNA methylation assay kit. **B** The m6A level of total RNA in different cell lines was determined by the m6A RNA methylation assay kit. **C** Relative m6A level of STAT3 mRNA was measured by MeRIP-qRT-PCR analysis in different cell lines. **D** Metagene profiles of m6A peaks distribution across the transcriptome in MHCC97H cells. **E** Distribution of m6A peaks in the 3'UTR (green), coding sequence (CDS, red), and 5'UTR (blue) of RNA transcripts. **F** The consensus sequence GAAC (RRACH) motif for m6A methylation was identified in MHCC97H cells. **G** The m6A abundance in STAT3 mRNA transcripts was detected by m6A-seq in MHCC97H cells. The detected m6A peaks were marked by a yellow box. **H**, **I** qRT-PCR analysis of MeRIP in MHCC97H (H) and HCCLM3 (I) cells using indicated PCR primers. **J** qRT-PCR analysis of STAT3 mRNA enrichment in anti-METTL3 immunoprecipitated RNA in MHCC97H and HCCLM3 cells. **K** MeRIP-qRT-PCR analysis of m6A levels of STAT3 in MHCC97L cells transfected with the indicated plasmids. **L** MeRIP-qRT-PCR analysis of m6A levels of STAT3 in MHCC97H cells transfected with the indicated siRNAs. **M** The diagram of pGL3-Control luciferase reporter constructs containing the fragment of human STAT3 3'UTR. The constructs of wild-type (WT), mutation (T-to-A mutation) of the m6A site (MUT), and deletion of the m6A site (DEL) were separately shown in the diagram. **N** Luciferase reporter gene assay of STAT3 3'UTR activities in HEK293T cells. The cells were transiently transfected with si-Control or si-METTL3#1 and the WT, MUT, or DEL constructs. All experiments were repeated at least three times. Error bars represent mean ± SD. *P < 0.05, **P < 0.01, ***P < 0.001 by 2-tailed Student’s *t-*test

The paired HCC tissues and non-tumor tissues used in Figs. 1H, I and 2A (*n* = 10) and Fig. S3C (*n* = 4) were collected from the patients at First Affiliated Hospital of Wenzhou Medical University. This study was evaluated and reviewed by the Ethics Committee of Wenzhou Medical University. Written consent was obtained from all patients prior to the study. The clinical characteristics of patients are presented in supplementary table S3.

### M6A assay in total RNA

Total RNA was extracted from corresponding tissues or cells using Trizol reagent (Invitrogen, USA) according to the manufacturer's instructions. Total RNA concentration was determined by NanoDrop 2000 (Thermo, USA). According to the standard protocol of m6A RNA Methylation Assay Kit (# ab185912, Abcam), the RNA was incubated with m6A capture antibody solution in each well for 1 h. After incubation, the m6A capture antibody solution was removed and the well was washed by wash buffer for three times. Similarly, the detection antibody was added for incubation (30 min) and then removed. After washing by wash buffer, the diluted enhancer solution was added and incubated for 30 min. Removed the diluted enhancer solution and washed. Finally, the absorbance was read at 450 nm after the addition of developer solution and stop solution. According to the manufacturer's requirements, the m6A content was calculated based on the standard curve.

### Methylated RNA immunoprecipitation (MeRIP) and m6A sequencing (m6A-seq)

The methylated RNA immunoprecipitation (MeRIP) was performed according to a previously described protocol [23]. In brief, the total RNA was extracted from cells using Trizol reagent (Invitrogen, USA), and mRNA was purified from the total RNA by Gen Elute mRNA Miniprep Kit (Sigma-Aldrich) according to the manufacturer's instructions. NanoDrop 2000 was used to measure the concentration of mRNA. Then, the purified mRNA was incubated with anti-m6A antibody (#202,003, Synaptic Systems) accompanied with the mixture of Protein A beads (Thermo Fisher). After incubation, the bound RNA was washed and eluted twice by elution buffer containing the m6A 5’-monophosphate sodium salt (Sigma-Aldrich). Then, the RNA was extracted and purified by phenol–chloroform. The RNA reverse transcription kit (TaKaRa, Japan) was used for cDNA synthesis. Finally, qRT-PCR was performed to examine m6A enrichment. The related primers are listed in supplementary table S4. For the m6A sequencing, the library mRNAs (fragmented into ~ 100nt and enriched by m6A antibody) and the input mRNAs were prepared using KAPA standard mRNA-seq kit (Illumina). The Clustered libraries were loaded onto the reagent cartridge and forwarded to the sequencing run on Illumina Hiseq 4000 system. Library preparation and high-throughput sequencing were performed by Aksomics Inc. (Shanghai, China).

### Transwell assay and Wound healing assay

For transwell assay, a polycarbonate transwell chamber (#3422, Corning, USA) with an 8-μm pore size was used to evaluate the migration and invasion of indicated cells. For the invasion assay, the matrigel (#354,234, BD Biosciences, USA) was spread on the upper chamber. 5 × 10^4^ cells were seeded into the upper chamber with 150 µl medium without FBS (two replicates per sample). The chambers were then placed in the medium with 18% FBS for 48 h to drive cell invasion. Cells were removed from the upper chamber with a cotton swab, and invasive cells in the lower chamber were fixed in 4% paraformaldehyde and stained with 0.5% crystal violet. For the migration assay, the experiment was performed with the same method but without supplementation of matrigel in the upper chamber. Chambers were photographed, and total invasive (/migrated) cells were quantified by Image J software. For wound healing assays, cells cultured in 6-well plates were scratched by the tip of a small 200 µl pipette to create a "wound" and monitored for "healing" at indicated time points. The closure area was quantified by Image J software.

### Xenograft and in vivo imaging system analysis

Six-week-old male BALB/C athymic nude mice were purchased from Beijing Vital River Laboratory Animal Technology Co. Ltd. The mice were housed according to the guidelines of the National Institutes of Health Guide for the Care and Use of Laboratory Animals. The corresponding cells (5 × 10^6^) with luciferase signal were prepared and resuspended in 150 µl PBS/Matrigel (BD Biosciences, 1:1 mixture). The mice were randomly divided into 3 groups (*n* = 5 per group). The cells were injected into the mice's hepatic lobe. After 2 weeks, the mice were anesthetized with isoflurane and intraperitoneally injected with 150 mg/kg D-Luciferin once a week to evaluate tumor metastasis. D-Luciferin was obtained from Keyuandi Biotech Co. Ltd (Shanghai, China). The IVIS system was used to analyze the bioluminescence of mice. For direct assessment of lung metastasis, the lower body of mice was covered with black cardboard. After 7 weeks of injection, the nude mice were sacrificed by cervical dislocation. The weight of the tumors was recorded. After paraffin embedding, tumor samples were used for IHC and HE staining. In this experiment, researchers who conducted measurements were blinded to avoid biases. All animal experiments were approved by the Ethics Committee of Xinxiang Medical University for medical laboratory animal sciences.

### Statistical analysis

Statistical significance was assessed using GraphPad Prism 8. The values in the graphs represent the mean of three biologically independent experiments. Error bars represent mean ± SD. The significance was set as *P < 0.05, **P < 0.01, ***P < 0.001 by 2-tailed Student’s t-test. For the partial analysis of in vitro and in vivo experiments, the significance was assessed by one-way ANOVA. The correlations between METTL3 expression with the overall survival and relapse-free survival were separately analyzed via the online resource GEPIA (http://gepia.cancer-pku.cn/) and Kaplan–Meier Plotter (https://kmplot.com/analysis/index.php?cancer=liver_rnaseq&p=service). The correlation of gene expression in HCC tissue microarray was analyzed by Spearman correlation analysis. The statistics of IHC staining and nuclear fluorescence were assessed by image J.

## Results

### The expression of STAT3 is positively related to METTL3 in HCC

First, we assessed the relationship between METTL3 expression and the survival of HCC patients by online analysis tools GEPIA [24] and Kaplan–Meier Plotter [25]. The results revealed that higher expression of METTL3 correlated with shorter survival times (Fig. 1A, B), suggesting the potential role of METTL3 on HCC progression. STAT3 is a classic oncogene that plays a crucial role in promoting the proliferation and metastasis of cancer cells [19, 26, 27]. In a previous study, we revealed the pro-metastatic effect of STAT3 on HCC cells [19]. Thus, we wondered if there was a potential relationship between METTL3 and STAT3 in the promotion of HCC metastasis. To test this conjecture, we quantified the expression level of STAT3 and METTL3 by Western blotting assay. Compared with MHCC97L and HepG2 cell lines, METTL3 and STAT3 were more strongly expressed in the high-metastatic MHCC97H and HCCLM3 cell lines (Fig. 1C). A microarray involving 122 clinical HCC tissues was used for immunohistochemistry (IHC) staining of METTL3 and STAT3. The results indicated that STAT3 was positively correlated to METTL3 in the examined HCC specimens (Fig. 1D, E; spearman correlation analysis, *r* = 0.656, *p* < 0.001). Notably, strong staining of METTL3 and STAT3 was observed in metastatic tissues compared with non-metastatic tissues (Fig. 1F, G), suggesting the potentially vital role of METTL3 and STAT3 in cell metastasis. Moreover, Western blotting analysis indicated that METTL3 and STAT3 were highly expressed in HCC tissues in comparison with paired non-tumor tissues (*n* = 10) (Fig. 1H, I). These findings reveal that the expression of STAT3 is positively related to METTL3 in HCC.

### METTL3 mediates m6A modification of STAT3 mRNA in HCC cells

Given the close association between METTL3 and STAT3, we sought to explore the regulatory mechanism between them in HCC cell metastasis. First, we evaluated the m6A level of total RNA in paired HCC tissues and corresponding non-tumor tissues (*n* = 10). The m6A levels in HCC tissues were upregulated compared to non-tumor tissues (Fig. 2A). Moreover, the m6A levels in high-metastatic HCC cell lines MHCC97H and HCCLM3 were higher than those in low-metastatic MHCC97L and HepG2 and normal liver L-O2 cells (Fig. 2B). These results indicated the association between high m6A levels and cell metastasis. As shown in Fig. 1C, higher STAT3 expression levels were observed in high-metastatic MHCC97H and HCCLM3 cell lines. Thus, we hypothesized whether m6A modification was involved in regulating STAT3 expression. Methylated RNA immunoprecipitation (MeRIP) analysis showed that STAT3 mRNA was indeed subjected to m6A modifications. Notably, the m6A levels of STAT3 mRNA in MHCC97H and HCCLM3 were higher than those in MHCC97L and L-O2 cells (Fig. 2C). To localize the m6A site of STAT3 at a transcriptome-wide level, we performed m6A sequencing (m6A-seq) using mRNA isolated from MHCC97H cells. In line with previous studies, the m6A peaks were mostly enriched in the CDS and 3’-untranslated region (UTR) (Fig. 2D, E). The m6A consensus sequence GAAC (RRACH) motif was identified to be highly enriched within m6A sites in the immunopurified RNA (Fig. 2F). M6A-seq data showed that STAT3 mRNA had two m6A peaks, both of which were in the 3'UTR of STAT3 mRNA, and the positions of these two peaks almost overlapped (peak1:40,466,894–40,467,104; peak2:40,466,931–40,467,111, the details were presented in Supplementary table S5). Then, we visualized the sequencing results and found the m6A peak according to the location information (Fig. 2G). Subsequently, we performed MeRIP and qRT-PCR to validate the m6A-seq data in STAT3 mRNA (Fig. 2H, I). Different primers were used to amplify the m6A peak region or a control (non-peak) region of STAT3 mRNA. Meanwhile, EGFR (a methylated mRNA) and RCN2 (a non-methylated mRNA) were separately amplified as positive control and negative control. The results confirm the presence of the m6A peak in STAT3 mRNA (Fig. 2H, I).

To investigate the potential effect of METTL3 on the m6A modification of STAT3, we performed RIP assays in MHCC97H and HCCLM3 cells. The data revealed the interaction between METTL3 and STAT3 mRNA (Fig. 2J). Moreover, the m6A level of STAT3 was upregulated in METTL3-overexpressed MHCC97L cells relative to control cells (Fig. 2K). Decreased m6A level of STAT3 was also observed in METTL3-knockdown MHCC97H cells compared to control cells (Fig. 2L). Next, we performed dual-luciferase reporter and mutagenesis assays in HEK293T cells (Fig. 2M). The data revealed that knockdown of METTL3 obviously depressed the luciferase activity of reporter carrying wild-type 3'UTR fragment of STAT3 (Fig. 2N). However, this phenomenon was disappeared when the RRACH motif was deleted or mutated in the abovementioned m6A peak (Fig. 2N), suggesting that METTL3 induces m6A modification of STAT3 mRNA at this site. Overall, the above results support that METTL3 induces the m6A modification of STAT3 in HCC cells.

### METTL3 upregulates STAT3 by promoting the translation of STAT3 mRNA

**Fig. 3:**
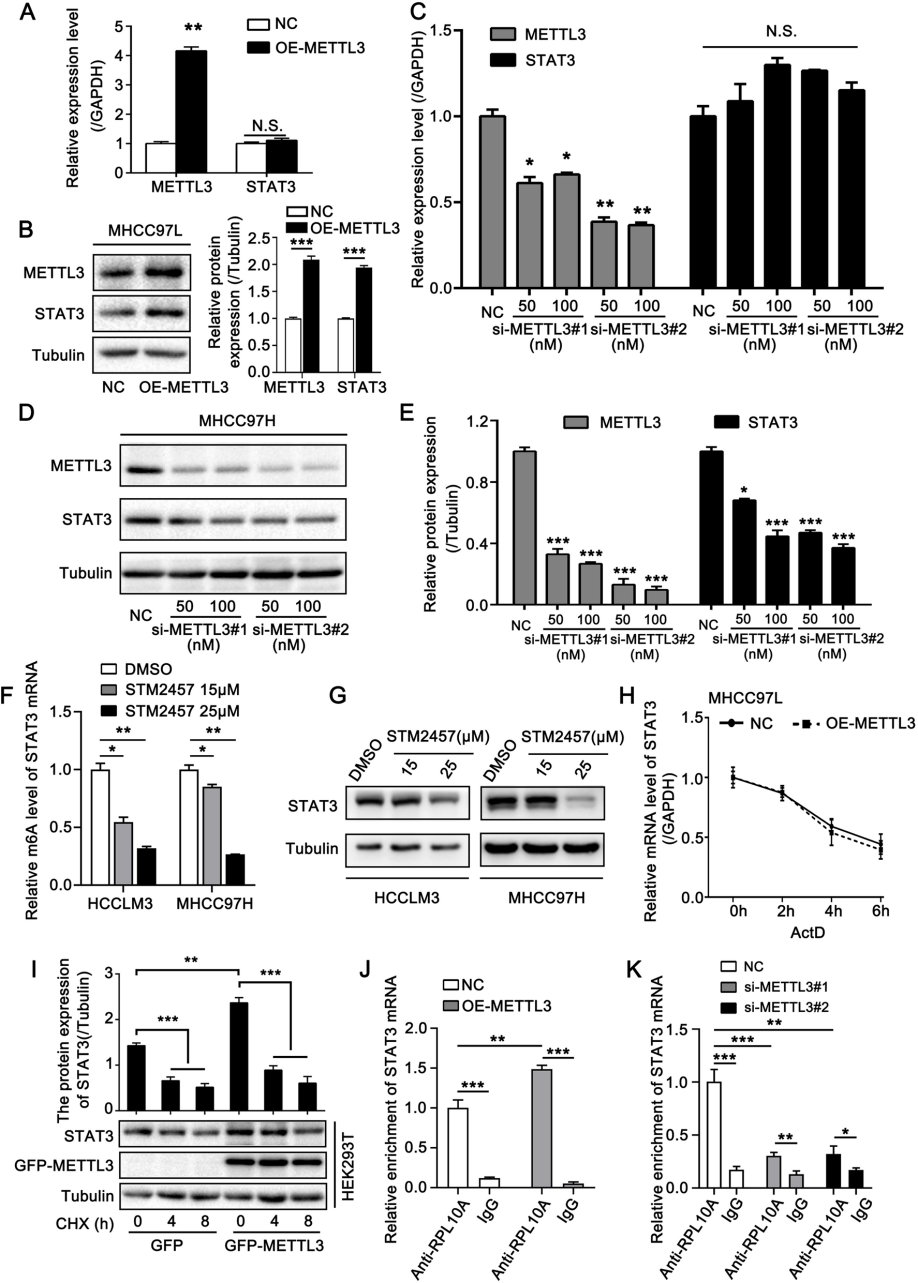
METTL3 upregulates STAT3 by promoting the translation of STAT3 mRNA. **A**, **B** qRT-PCR (A) and Western blotting analysis (B) of METTL3 and STAT3 in MHCC97L cells transfected with the indicated plasmids. The right panel in (B) shows the quantification of the intensity relative to tubulin. **C** qRT-PCR analysis of METTL3 and STAT3 in MHCC97H cells transfected with the indicated siRNAs. **D**, **E** Western blotting analysis of METTL3 and STAT3 in MHCC97H cells transfected with si-Control or si-METTL3. (E) shows the quantification of the intensity relative to tubulin. **F** MeRIP-qRT-PCR analysis of m6A levels of STAT3 in HCCLM3 and MHCC97H cells treated with the indicated concentration of STM2457 for 48 h. **G** Western blotting analysis of STAT3 in HCCLM3 and MHCC97H cells treated with the indicated concentration of STM2457 for 48 h. **H** qRT-PCR analysis of STAT3 mRNA stability in MHCC97L cells upon treatment with transcription inhibitor Actinomycin-D (ActD) for the indicated timepoints. The cells were transfected with empty vector or METTL3. **I** Western blotting analysis of STAT3 in HEK293T cells time-dependently treated with 100 μg/ml cycloheximide (CHX) after being transiently transfected with the indicated plasmids. The upper panel is the quantification of the intensity of STAT3 relative to tubulin. **J** qRT-PCR analysis of STAT3 mRNA enrichment in anti-RPL10A immunoprecipitated RNA in MHCC97L cells transfected with the indicated plasmids. **K** qRT-PCR analysis of STAT3 mRNA enrichment in anti-RPL10A immunoprecipitated RNA in MHCC97H cells transfected with the indicated siRNAs. All experiments were repeated at least three times. Error bars represent mean ± SD. *P < 0.05, **P < 0.01, ***P < 0.001 by 2-tailed Student’s *t-*test

Next, we explored whether METTL3-induced m6A modification affected STAT3 expression. qRT-PCR and Western blotting analyses were performed in different cell lines. Overexpression of METTL3 in MHCC97L cells upregulated the protein levels of STAT3 while mRNA levels were not altered (Fig. 3A, B). Meanwhile, silencing of METTL3 decreased the protein levels of STAT3, but no changes were observed at the mRNA level (Fig. 3C-E, Fig. S1A). Next, we treated cells with STM2457, a METTL3-specific inhibitor, and detected the changes in STAT3 m6A level. We found that the m6A level of STAT3 mRNA was reduced upon STM2457 treatment (Fig. 3F). It was worth noting that treatment of STM2457 also caused the decrease of STAT3 protein level, however, the mRNA level had no significant alteration (Fig. 3G, Fig. S1B, C). Moreover, METTL3 overexpression did not affect the stability of STAT3 mRNA (Fig. 3H, Fig. S1D). These results indicated that METTL3 might regulate the translation or protein stability of STAT3. To assess whether METTL3 modulated STAT3 protein stability, we treated cells with cycloheximide (CHX, a protein translation inhibitor) to block translation and measured STAT3 levels. The results showed that overexpression of METTL3 yielded no significant stabilizing effect on STAT3 protein levels (Fig. 3I). Therefore, we hypothesized that METTL3 could regulate the translation of STAT3 mRNA. Rodrigues et al. have reported that the ribosome-engaged RNA fraction is a reflection of the translation activity of gene [28]. They used GFP-tagged RPL10A to track the translating mRNA [28]. Thus, we performed RIP assay and found that METTL3 overexpression enhanced the abundance of RPL10A-enriched STAT3 mRNA (Fig. 3J); meanwhile, knockdown of METTL3 inhibited the enrichment of RPL10A on STAT3 mRNA (Fig. 3K).

Next, we investigated the impacts of translation-associated m6A reader proteins, including YTHDF1/3, YTHDC2, and IGF2BP1/2/3 on the expression of STAT3. We observed that both of them had no obvious effect on STAT3 expression level (Fig. S1E-L). Interestingly, Gregory et al. reported that METTL3 could recruit eIF3 to the translation initiation complex and facilitate the translation of m6A-contained mRNA independent of methyltransferase activity or downstream m6A reader proteins [29]. eIF3b is the core subunit of the translation initiation factor eIF3 [29]. We performed a RIP assay and found that overexpression of wild-type of METTL3 and catalytic mutant METTL3 both effectively enhanced eIF3b-immunoprecipitated STAT3 mRNA (Fig. S1M, N). However, the catalytic mutant METTL3 could not upregulate the protein level of STAT3 while the wild-type of METTL3 did (Fig. S1O). This data was consistent with the previous reports, which showed that catalytic mutant METTL3 could interact with the translation initiation machinery but not elevate the protein level of target genes [30]. The above data indicated that the methyltransferase activity of METTL3 was indispensable for the upregulation of STAT3.

### STAT3 regulates the nuclear localization of METTL3 via WTAP

**Fig. 4:**
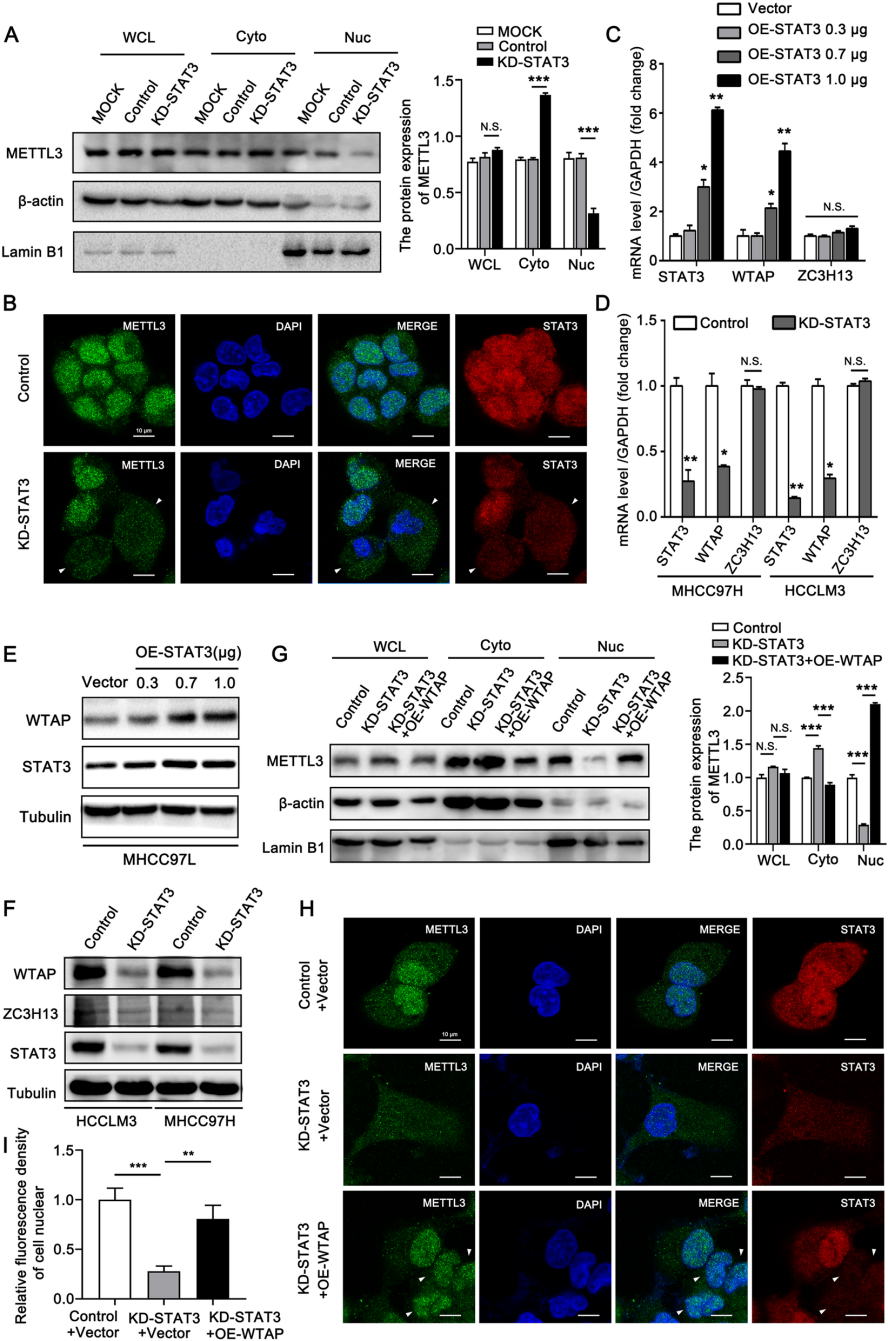
STAT3 regulates the nuclear localization of METTL3 via WTAP. **A** Western blotting analysis of METTL3 in the whole-cell lysate (WCL), cytoplasmic (Cyto), and nuclear (Nuc) fractions from the HCCLM3 (MOCK), HCCLM3-knockdown-control (Control), and HCCLM3-STAT3-knockdown (KD-STAT3) cells. β-actin and Lamin B1 were used as cytoplasmic and nuclear markers, respectively. The right panel shows the quantification of the intensity relative to β-actin or Lamin B1. **B** Immunofluorescence analysis of METTL3 (green), STAT3 (red) and DAPI (blue) in HCCLM3-STAT3-knockdown and HCCLM3-control cells. Scale bar, 10 μm. **C** qRT-PCR analysis of STAT3, WTAP, and ZC3H13 in MHCC97L cells transfected with the indicated plasmids. **D** qRT-PCR analysis of STAT3, WTAP and ZC3H13 in STAT3 knockdown and control MHCC97H cells, STAT3 knockdown and control HCCLM3 cells. **E** Western blotting analysis of WTAP and STAT3 in MHCC97L cells transfected with the indicated plasmids. **F** Western blotting analysis of WTAP, ZC3H13, and STAT3 in STAT3 knockdown and control MHCC97H cells, STAT3 knockdown and control HCCLM3 cells. **G** Western blotting analysis of METTL3 in the whole-cell lysate (WCL), cytoplasmic (Cyto), and nuclear (Nuc) fractions from the HCCLM3-knockdown-control, HCCLM3-STAT3-knockdown, and HCCLM3-STAT3-knockdown-WTAP-overexpression cells. β-actin and Lamin B1 were used as cytoplasmic and nuclear markers, respectively. The right panel shows the quantification of the intensity relative to β-actin or Lamin B1. **H, I** Immunofluorescence analysis of METTL3 (green), STAT3 (red) and DAPI (blue) in the indicated cells. Scale bar, 10 μm. (I) shows the quantification of nuclear fluorescence intensity of anti-METTL3 cell. All experiments were repeated at least three times. Error bars represent mean ± SD. *P < 0.05, **P < 0.01, ***P < 0.001 by 2-tailed Student’s *t-*test

Given the significant association between METTL3 and STAT3 in HCC, we explored whether STAT3 played a potential role in modulating the expression and/or function of METTL3. qRT-PCR and Western blotting analysis showed that silencing STAT3 did not affect the expression of METTL3 (Fig. S2A, B). Previous studies corroborate that METTL3 is primarily localized in nuclear speckle, convenient for the methyltransferase activity of METTL3 in regulating the splicing and export of mRNA [31, 32]. Thus, we investigated the impact of STAT3 on the cellular distribution of METLL3. The nuclear and cytosolic fractions were analyzed in the stable-knockdown-STAT3 cells and control cells. STAT3 depletion decreased the nuclear level of METTL3 and increased its cytosolic level to a certain extent, while the overall protein level of METTL3 was not altered (Fig. 4A). Immunofluorescence staining showed that compared with the non-deleted cells, the nuclear localization of METTL3 was decreased in the STAT3-deleted cells (Fig. 4B, Fig. S2C), suggesting that STAT3 might modulate the cellular distribution of METTL3 by interacting with METTL3. However, the Co-IP assay displayed that STAT3 could not interact with METTL3 (Fig. S2D, E). WTAP and ZC3H13 have been reported to modulate the cellular distribution of METTL3 [31, 33]. Yang et al. found that WTAP could interact with METTL3 and was responsible for nuclear speckle localization, essential for methyltransferase activity of METTL3 [31]. Diao et al. showed that ZC3H13, a zinc-finger protein, played a crucial role in anchoring WTAP in the nucleus to modulate RNA m6A modification [33]. We examined the effect of STAT3 on WTAP and ZC3H13 expression levels. STAT3 overexpression and knockdown significantly upregulated and downregulated WTAP expression, but the expression levels of ZC3H13 were unaltered (Fig. 4C-F, Fig. S2F, G). Thus, we speculated that WTAP might be involved in STAT3-modulated nuclear localization of METTL3. Notably, subcellular fractionation and immunofluorescence staining analyses both demonstrated that overexpression of WTAP could prevent nuclear export of METTL3 induced by STAT3 depletion (Fig. 4G-I). Collectively, STAT3 regulates the nuclear localization of METTL3 via WTAP.

### STAT3 upregulates WTAP expression by stimulating its transcription

**Fig. 5:**
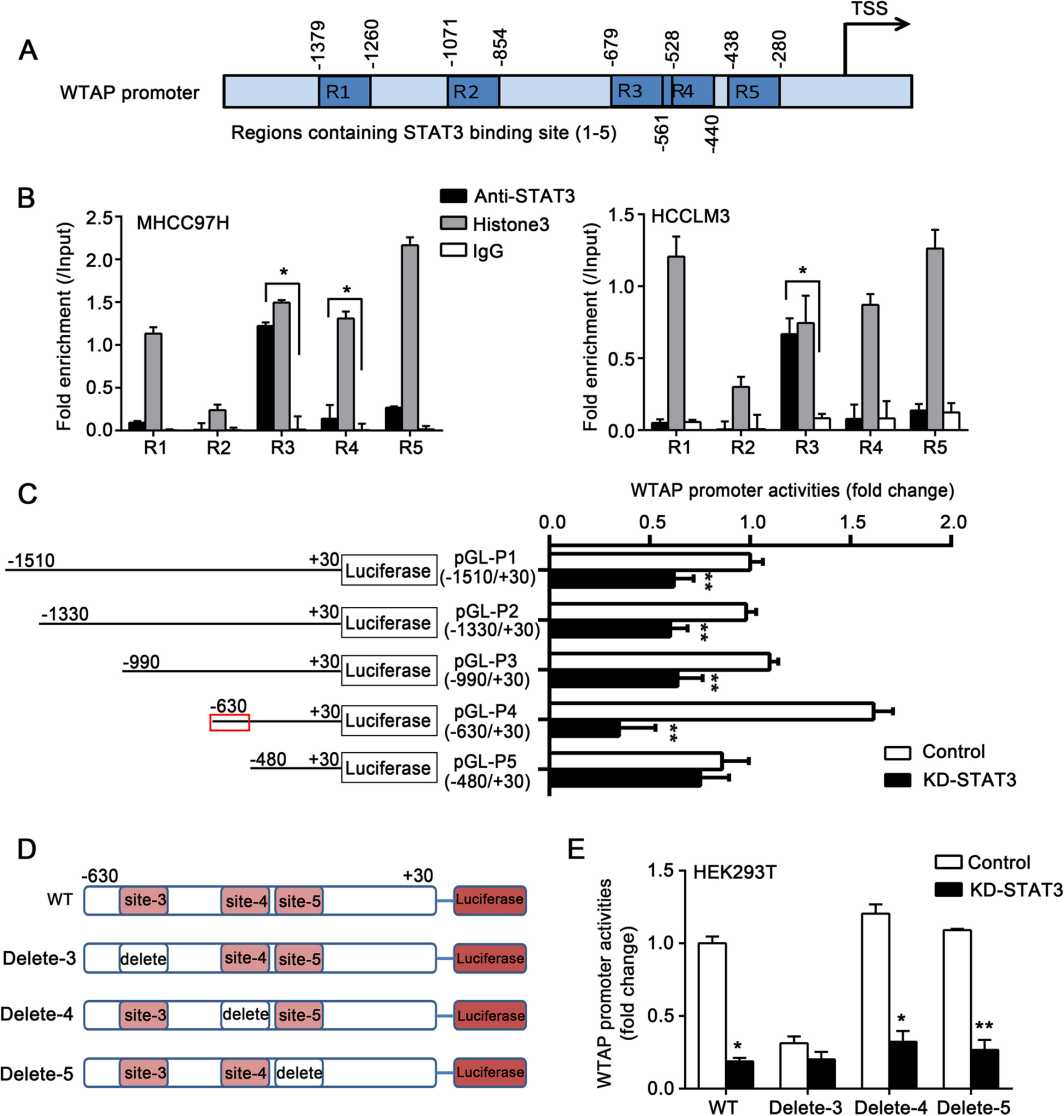
STAT3 upregulates WTAP expression by stimulating its transcription. **A** The diagram shows the five regions which contain the binding sites of STAT3 in the WTAP promoter. **B** qRT-PCR analysis of the enrichments of five regions in anti-STAT3, Histone3, and IgG immunoprecipitated DNA in MHCC97H (left panel) and HCCLM3 (right panel) cells. **C** Activities of corresponding fragments of WTAP promoter were examined by luciferase reporter gene assay in HEK293T cells. The cells were transiently transfected with control siRNA or si-STAT3#1 along with indicated fragments of WTAP promoter. **D** The diagram of pGL3-basic luciferase reporter constructs containing the fragment of human WTAP promoter (pGL-P4). The constructs of wild-type (WT), Delete-3 (the binding site3 (-602 ~ -595) was deleted), Delete-4 (the binding site4 (-454 ~ -447) was deleted), and Delete-5 (the binding site5 (-438 ~ -430) was deleted) were separately shown in the diagram. **E** Luciferase reporter gene assay of WTAP promoter activities in HEK293T cells. The cells were transiently transfected with control siRNA or si-STAT3#1 and the WTAP promoter (WT) or constructs with deleted binding sites of STAT3. All experiments were repeated at least three times. Error bars represent mean ± SD. *P < 0.05, **P < 0.01, ***P < 0.001 by 2-tailed Student’s *t-*test

Given the effect of STAT3 on WTAP expression, we further explored whether STAT3 could act directly upon the promoter of WTAP to stimulate its transcription. Online resources (http://gpminer.mbc.nctu.edu.tw/, http://gene-regulation.com/pub/programs.html) were used to analyze the binding site of STAT3 on WTAP promoters. Five putative binding sites of STAT3 were identified on the promoter of WTAP. The ChIP assay was performed, and the specific primers were used to amplify the five regions containing the corresponding binding sites. The data showed that STAT3 could occupy the promoter of WTAP at region 3 (Fig. 5A, B). Then, we cloned a series of fragments of WTAP promoter (Fig. 5C). The luciferase reporter gene assay revealed that the activity of pGL-P4 was highest among the five fragments of WTAP promoter. Knockdown of STAT3 efficiently suppressed the activities of WTAP promoter except for pGL-P5 (Fig. 5C). This data suggested that the region (-630 ~ -480) of the WTAP promoter might contain the core sequence modulated by STAT3 (Fig. 5C). Notably, region 3 (-679 ~ -528) assessed by the ChIP assay was overlapped with the (-630 ~ -480) region confirmed by the luciferase reporter assay, indicating the significance of site 3 (-602 ~ -595) in activating the WTAP promoter. Next, we constructed the site3-deletion mutant based on pGL-P4 and performed a luciferase reporter gene assay. Site 4 (-454 ~ -447) and site 5 (-438 ~ -430) were separately deleted as negative controls (Fig. 5D). Compared with the wild-type (WT), the activity of Delete-3 significantly decreased. Moreover, the activity of Delete-3 was not altered in response to STAT3 knockdown, while Delete-4 and Delete-5 activities were significantly suppressed upon STAT3 knockdown (Fig. 5E). These findings reveal that STAT3 stimulates WTAP transcription via binding to site 3.

Furthermore, tissue microarray analysis uncovered a positive relationship between the expression levels of STAT3 and WTAP in cancer tissues and adjacent non-cancerous tissues (Fig. S3A, B). Similarly, Western blotting analysis in 4 paired HCC tissue specimens also revealed higher levels of STAT3 and WTAP in HCC tissues compared with non-cancerous tissues (Fig. S3C). TCGA database analysis by UALCAN (http://ualcan.path.uab.edu/) showed that WTAP mRNA levels in HCC tissues were significantly elevated than in non-tumor tissues (Fig. S3D). In addition, WTAP expression was closely related to the tumor stage of HCC (Fig. S3E). Moreover, Kaplan–Meier analysis [25] uncovered that the patients with higher expression levels of WTAP conversely had a shorter survival time (Fig. S3F, G). Taken together, these results indicate that WTAP is overexpressed and correlated with STAT3 in HCC tissues.

### STAT3 modulates the methyltransferase function of METTL3 via WTAP

**Fig. 6:**
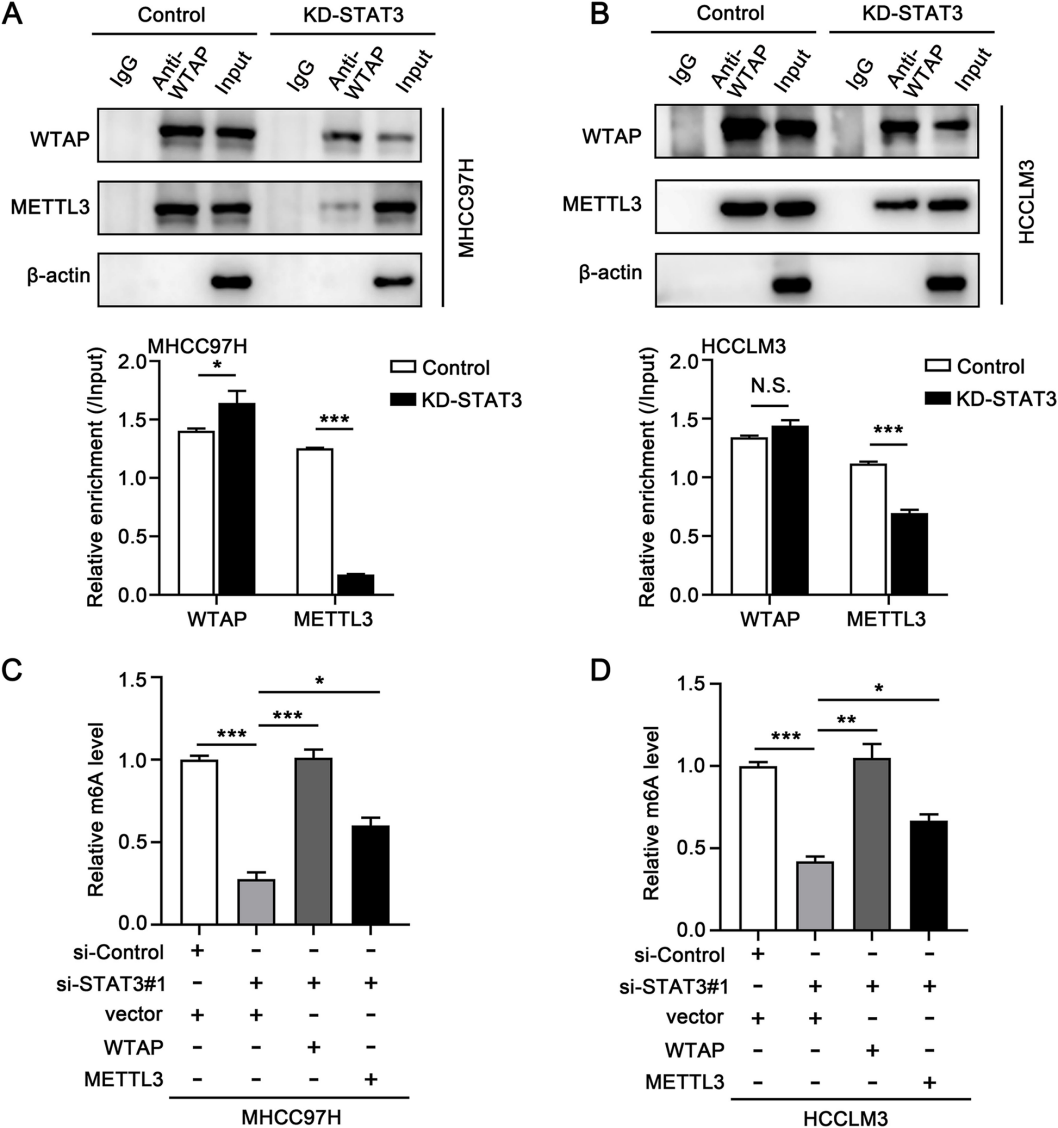
STAT3 modulates the methyltransferase function of METTL3 via WTAP. **A**, **B** Co-IP analysis of the interaction between METTL3 and WTAP in STAT3 knockdown and control MHCC97H cells (A), STAT3 knockdown and control HCCLM3 cells (B). The lower panels show the quantification of intensity relative to input. **C**, **D** The m6A level of total RNA in MHCC97H cells (C) and HCCLM3 cells (D) were determined by the m6A RNA methylation assay kit. The cells were transfected with the indicated siRNAs and/or plasmids. All experiments were repeated at least three times. Error bars represent mean ± SD. *P < 0.05, **P < 0.01, ***P < 0.001 by 2-tailed Student’s *t-*test

To further verify the relationship among STAT3, WTAP, and METTL3, we performed Co-IP assays in MHCC97H and HCCLM3 cells. Consistent with the previous study [31], we found that METTL3 could interact with WTAP (Fig. 6A, B). However, STAT3 depletion obviously diminished the interaction of WTAP with METTL3 by depressing WTAP expression (Fig. 6A, B). Next, we measured m6A levels in MHCC97H and HCCLM3 cells following STAT3 knockdown. The m6A level was downregulated upon STAT3 depletion (Fig. 6C, D). Interestingly, we observed that overexpression of WTAP efficiently alleviated STAT3 knockdown-induced decrease of m6A level, while METTL3 overexpression only alleviated this decrease to a certain extent (Fig. 6C, D). This might be because METTL3 overexpression could not compensate for the failure of nuclear speckle localization caused by WTAP deficiency. Overall, these data indicate that STAT3 modulates the methyltransferase function of METTL3 through WTAP.

### METTL3-STAT3 positive feedback loop promotes cell metastasis in vitro and in vivo

**Fig. 7:**
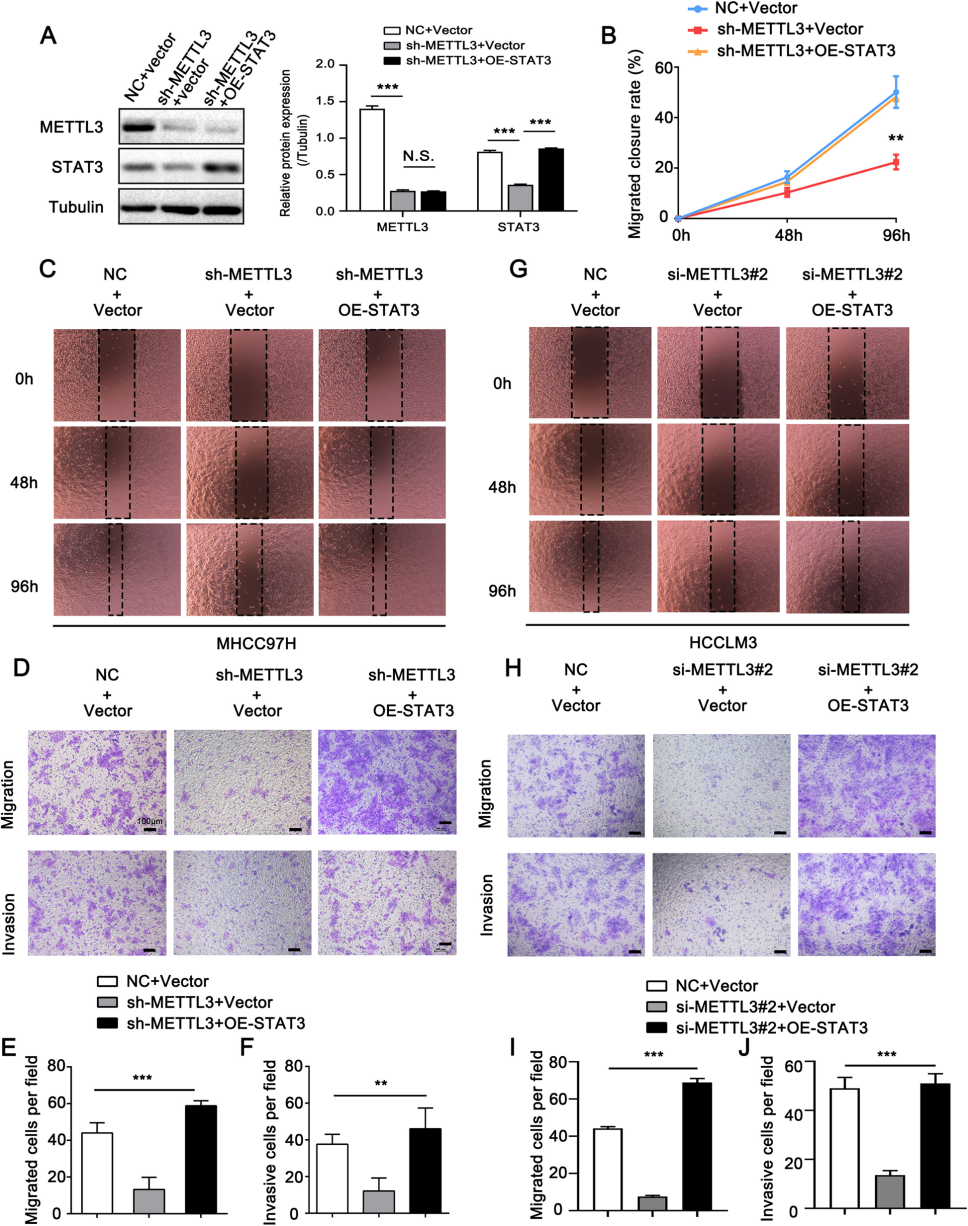
METTL3-STAT3 positive feedback loop promotes cell metastasis in vitro. **A** Western blotting analysis of METTL3 and STAT3 in the indicated cells shows the corresponding interfere/overexpress efficiency. The right panel shows the quantification of intensity relative to tubulin. Error bars represent mean ± SD, *P < 0.05, **P < 0.01, ***P < 0.001 by 2-tailed Student’s *t-*test. **B**, **C** Wound healing assay of the migration ability in the indicated cells. **D-F** Transwell assay of the migration and invasion abilities in the indicated cells. **G** Wound healing assay of the migration ability in HCCLM3 cells transfected with indicated siRNAs and plasmids. **H-J** Transwell assay of the migration and invasion abilities in HCCLM3 cells transfected with indicated siRNAs and plasmids. Error bars represent mean ± SD, *P < 0.05, **P < 0.01, ***P < 0.001 by one-way ANOVA

**Fig. 8:**
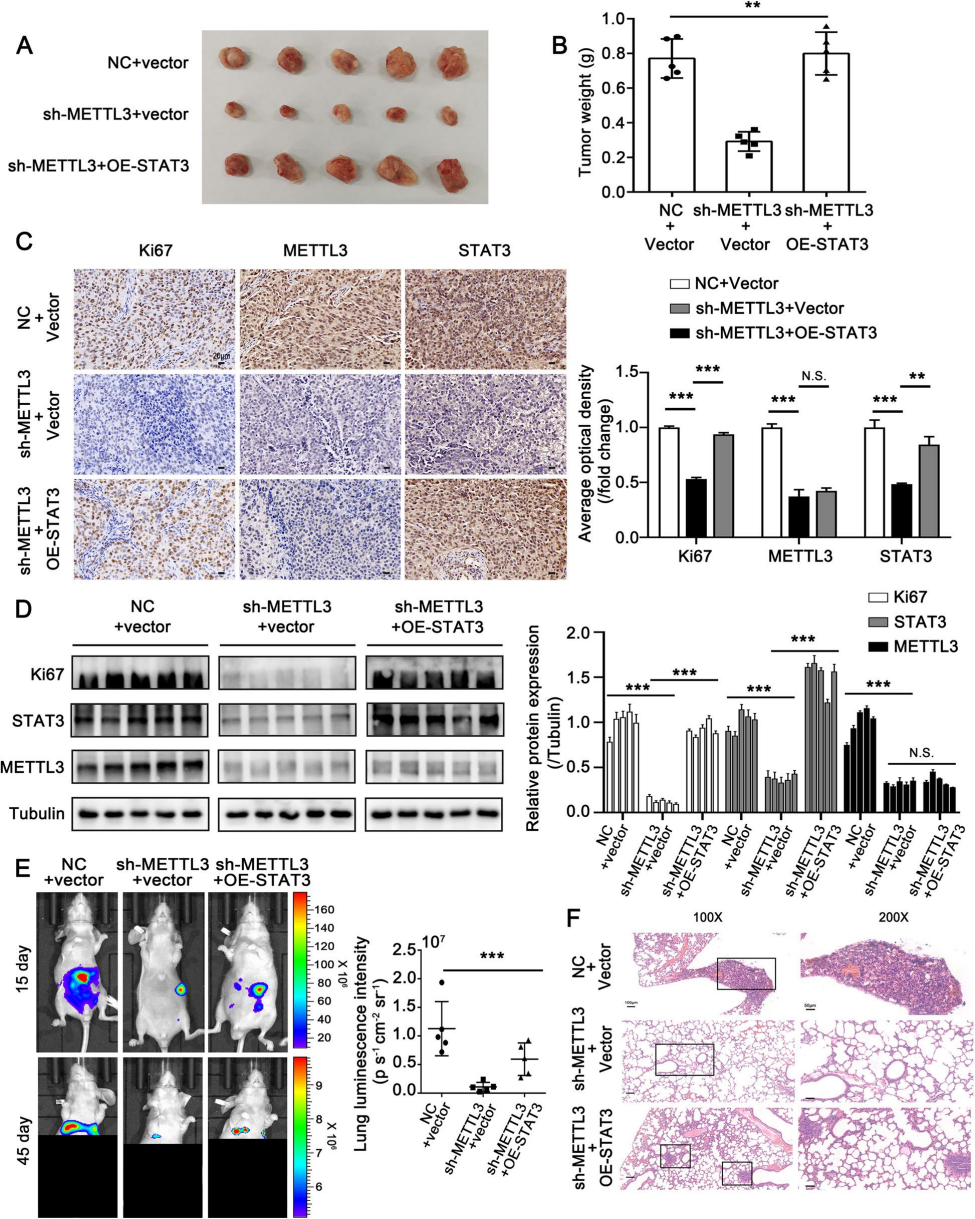
METTL3-STAT3 positive feedback loop promotes cell metastasis in vivo. **A**, **B** Imaging (A) and weights (B) of the xenograft tumors harvested from 5 mice per group. Error bars represent mean ± SD, *P < 0.05, **P < 0.01, ***P < 0.001 by one-way ANOVA. **C** IHC staining of Ki67, METTL3 and STAT3 in the tissues of xenograft tumors. Scale bar, 20 μm. The right panel shows the quantification of average optical density of Ki67, METTL3 and STAT3 in the tissues of xenograft tumors. *P < 0.05, **P < 0.01, ***P < 0.001 by 2-tailed Student’s *t-*test. **D** Western blotting analysis of the protein levels of METTL3, STAT3 and Ki67 in the indicated groups of tumor samples. The right panel shows the quantification of the intensity relative to tubulin. *P < 0.05, **P < 0.01, ***P < 0.001 by 2-tailed Student’s *t-*test. **E** The representative bioluminescence images were captured in the indicated time after transplantation. The lower panel shows the bioluminescence intensity (photons/s/cm^2^/sr) in the lungs was quantitatively analyzed. The significance of differences was assessed by one-way ANOVA, *P < 0.05, **P < 0.01, ***P < 0.001. **F** Representative images of hematoxylin–eosin (H&E) staining in lung tissue after transplantation

The above data revealed the comprehensive interplay between METTL3 and STAT3. They formed a positive feedback loop in HCC. Next, we intended to investigate the role of the feedback loop in HCC metastasis. Based on MHCC97H cells, we constructed different cell lines with luciferase signal, including stably depleted-METTL3, stably depleted-METTL3-overexpressed-STAT3, and corresponding control cells. The efficiencies of knockdown and overexpression were verified, as shown in Fig. 7A. The wound healing and transwell assays showed that METTL3 knockdown effectively inhibited the migration and invasive capacities of HCC cells, while overexpression of STAT3 countered the METTL3 depletion-induced inhibitory effect (Fig. 7B-F). Similarly, the functional assays in HCCLM3 cells after transient transfection showed consistent results as above (Fig. 7G-J). Then, we constructed an orthotopic xenograft model of HCC to evaluate the role of the METTL3-STAT3 feedback loop in HCC metastasis in vivo. The findings showed that the growth and weight of xenograft tumors were suppressed when METTL3 was silenced, and STAT3 overexpression could alleviate the METTL3 depletion induced-suppressive effect (Fig. 8A, B). Subsequently, we evaluated the expressions of proliferation marker Ki67, METTL3 and STAT3 in tumor tissues by IHC staining and Western blotting assay. IHC data uncovered that knockdown of METTL3 depressed the expression of Ki67 and STAT3. Meanwhile, STAT3 overexpression could rescue the reduced Ki67 expression caused by METTL3 knockdown (Fig. 8C, D). More importantly, lung metastasis was significantly reduced in mice with METTL3 knockdown compared to mice with control cells (Fig. 8E, F). Nonetheless, STAT3 overexpression could reverse METTL3 knockdown-induced reduction of lung metastasis (Fig. 8E, F). Overall, our findings suggest that the METTL3-STAT3 positive feedback loop promotes cell metastasis in HCC.

## Discussion

In this study, we analyzed the expressions of METTL3 and STAT3 in tissue microarray. The close relationship between METTL3 and STAT3 in primary and metastatic specimens suggested their potential role in promoting HCC metastasis. To clarify the regulatory mechanism between them, we first evaluated the modulatory role of METTL3 on STAT3 expression. A recent study showed that METTL3 enhanced JAK1 translation via a m6A-YTHDF1 axis, and subsequently promoted the activation of STAT3 [34]. Herein, we found that STAT3 was a direct target of METTL3-mediated m6A modification, and was upregulated by METTL3 in high-metastatic HCC cells.

Gregory's team have reported that METTL3 could interact with translation initiation machinery to facilitate m6A-modified mRNA translation independently of m6A readers [29, 30]. Moreover, the recruitment of translation initiation factors does not require the methyltransferase activity of METTL3, but its N-terminal eIF3h-interaction domain [30]. This is also confirmed by our study, both wild-type and catalytic mutant METTL3 can promote the enrichment of eIF3b (the core subunit of eIF3 complex) on STAT3 mRNA (Fig. S1M, N). Interestingly, although Gregory's team did not focus on this point, their data showed that the catalytic mutant of METTL3 could not upregulate the protein level of target genes, while wild-type METTL3 could [30]. This was consistent with our findings (Fig. S1O). Therefore, for METTL3, the C-terminal methyltransferase domain and N-terminal eIF3-interaction domain were both necessary in the upregulation of some oncogenic target genes and facilitating malignance. The effect of STM2457 treatment revealed by our data (Fig. 4F, G and Fig. S1B, C) and another study [35] further confirmed this conclusion.

Nuclear speckle localization is required for the interaction of METTL3 with its target mRNA [31, 32]. Herein, we provided evidence that STAT3 transcriptionally upregulated WTAP expression to promote the nuclear localization of METTL3. Deletion of STAT3 disrupted the interaction between WTAP and METTL3 and further impaired the methyltransferase function of METTL3. However, how WTAP regulated by STAT3 further affects the localization of METTL3, the molecular mechanism still needs to be further explored. First, besides the main research object METTL3, the cellular distributions of other MTC members like METTL14, WTAP, VIRMA and RBM15 can also be detected and analyzed, to explore whether STAT3-modulated WTAP indirectly affects the nuclear localization of METTL3 by modulating other members in MTC; Second, RNA is crucial for the recruitment and/or the nuclear speckle retention of the complex [31]. WTAP first binds to the substrate mRNA and then recruits METTL3/METTL14 to nuclear speckle [31, 36]. Therefore, it is worth paying attention to the impact of RNAase treatment on the rescue effect of WTAP. Third, besides WTAP, whether STAT3 can affect METTL3 localization by regulating other target genes or interacting with other proteins is still unknown.

In this study, we clarified the interplay between METTL3 and STAT3, which formed a positive feedback loop in HCC. To evaluate the role of this feedback loop in cell metastasis, in vitro and in vivo experiments were performed by knocking down METTL3 with or without STAT3 overexpression. STAT3 could alleviate the METTL3 depletion-induced inhibitory effect on cell metastasis, indicating that METTL3 could promote metastasis by acting on STAT3 in HCC. Notably, STAT3 could not totally reverse the METTL3 knockdown-induced inhibition on lung metastasis, which may be attributed to the fact that other target genes of METTL3 also play a role in cell metastasis. According to current reports, SOCS2, Snail, RDM1, and non-coding RNA LINC00958, etc. are all target genes of METTL3 in HCC; METTL3 could affect proliferation, migration, invasion and lipogenesis to facilitate HCC progression by regulating these target genes [15, 37–39]. Interestingly, SOCS2 is a negative regulator of JAK/STAT3 signaling pathway [40]. METTL3-induced decrease of SOCS2 is very likely to have a positive effect on STAT3 activation. Meanwhile, another study has revealed the mechanism of STAT3 crosstalk with the Snail-Smad3/TGF-β1 signaling pathways that synergistically facilitate EMT and migration in HCC [41]. It can be seen that there is also complex crosstalk between these target genes. Moreover, unlike other target genes, we found that STAT3 could in turn affect the methyltransferase function of METTL3 by regulating its localization, suggesting that STAT3 may also have effects on other target genes of METTL3.

In addition, we found that WTAP was closely associated with STAT3 expression in HCC tissues. Since STAT3 and METTL3 exhibited a pro-metastatic role in HCC, as a part of the feedback loop, we explored whether WTAP yielded a similar effect. As expected, WTAP overexpression enhanced the migration and invasive capacities of HCC cells (Fig. S4). However, in Fig. 8, we did not evaluate the potential effect of WTAP overexpression on cell metastasis after METTL3 deletion. WTAP mainly affected the cell localization of METTL3. Therefore, we infer that it is of little significance to use WTAP overexpression to rescue the metastasis inhibition induced by METTL3 knockdown.

**Fig. 9:**
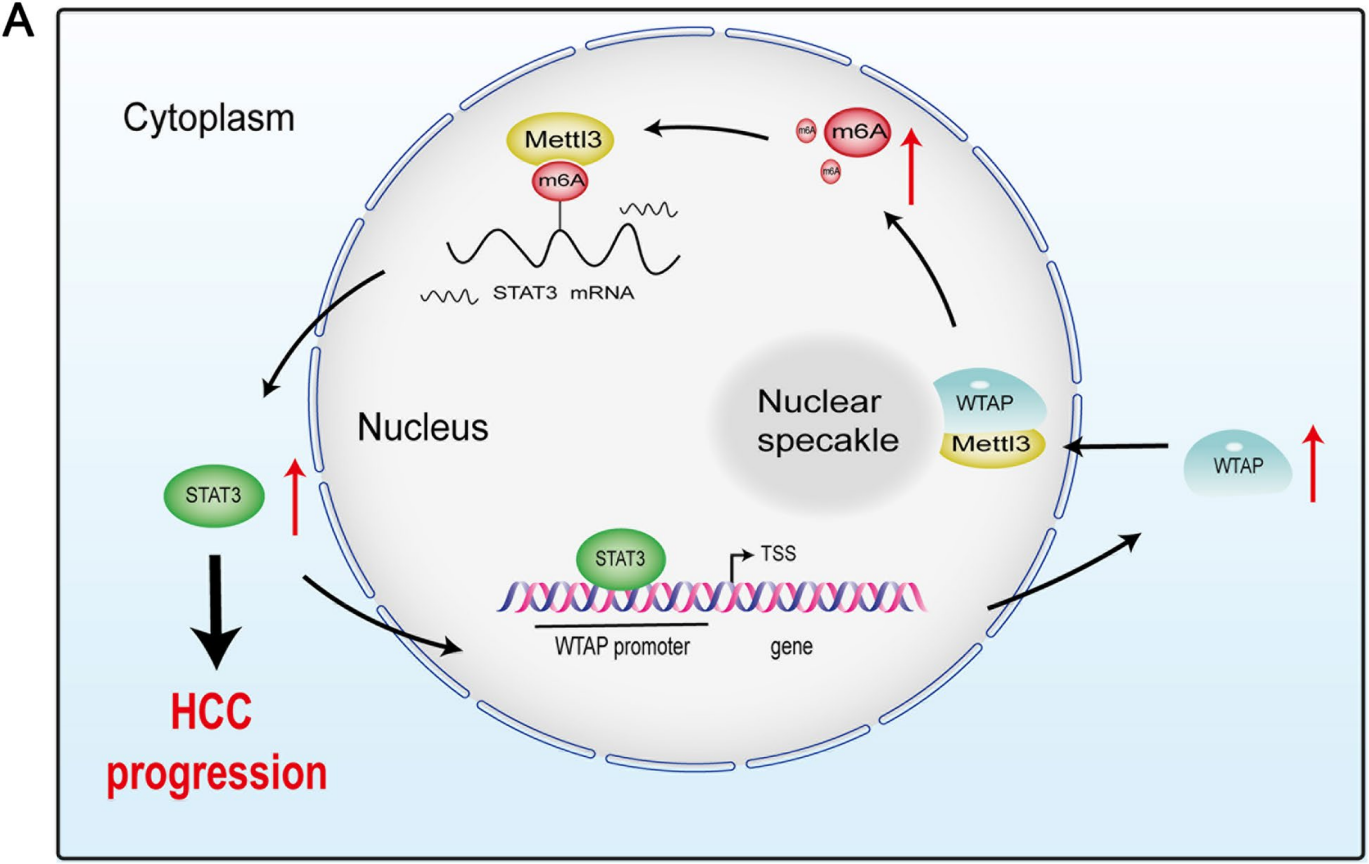
Working model of METTL3-STAT3 positive feedback loop in promoting HCC metastasis. METTL3 induces m6A modification to enhance STAT3 translation. Upregulated STAT3 interacts with WTAP promoter and stimulates the transcription of WTAP. Elevated WTAP promotes the nuclear localization of METTL3, facilitating m6A modification of target genes, including STAT3. Finally, they form a positive feedback loop to constantly enhance the expression of STAT3, thereby facilitating cell metastasis in HCC

In summary, we documented a new mechanism of HCC metastasis (Fig. 9) whereby METTL3 induces m6A modification to enhance STAT3 translation. Upregulated STAT3 interacts with WTAP promoter and stimulates the transcription of WTAP. Elevated WTAP promotes the nuclear localization of METTL3, facilitating m6A modification of target genes, including STAT3. Finally, they form a positive feedback loop to constantly enhance the expression of STAT3, thereby facilitating cell metastasis in HCC.

## Supplementary Information

**Additional file 1.**

**Additional file 2.**

**Additional file 3.**

**Additional file 4.**

**Additional file 5.**

**Additional file 6.**

## Data Availability

All data generated or analyzed during this study are included in this published article and its supplementary information files.
