# Supplementary material for: METTL3 and STAT3 form a positive feedback loop to promote cell metastasis in hepatocellular carcinoma
Source: Cell Commun Signal. 2023 May 25;21:121. doi: 10.1186/s12964-023-01148-7 (PMC10210303; doi:10.1186/s12964-023-01148-7)
Supplement: Supplementary file 4 — Additional file 3. [file 12964_2023_1148_MOESM3_ESM.doc]

**Supplementary Table S3.** Clinical characteristics of patients with liver cancer tissue specimens.

| NO. | Sex | Age | Organ | Pathology diagnosis | T | N | M | TNM | Stage |
| --- | --- | --- | --- | --- | --- | --- | --- | --- | --- |
| 1 | M | 63 | Liver | Hepatocellular carcinoma | T3 | N0 | M1 | T3N0M1 | IV |
| 2 | F | 53 | Liver | Hepatocellular carcinoma | T1 | N0 | MO | T1N0M0 | I |
| 3 | M | 76 | Liver | Hepatocellular carcinoma | T1 | NX | M0 | T1NXM0 | I |
| 4 | M | 45 | Liver | Hepatocellular carcinoma | T2 | N0 | M1 | T2N0M1 | IV |
| 5 | M | 72 | Liver | Hepatocellular carcinoma | T2 | N0 | M1 | T2N0M1 | IV |
| 6 | M | 27 | Liver | Hepatocellular carcinoma | T3 | N0 | M3 | T3N0M2 | IV |
| 7 | M | 49 | Liver | Hepatocellular carcinoma | T2 | N0 | M0 | T2N0M0 | II |
| 8 | M | 65 | Liver | Hepatocellular carcinoma | T2 | N0 | M0 | T2N0M0 | II |
| 9 | M | 67 | Liver | Hepatocellular carcinoma | T3 | N0 | M1 | T3N0M1 | IV |
| 10 | M | 69 | Liver | Hepatocellular carcinoma | T2 | N0 | M1 | T2N0M1 | IV |

| NO. | Sex | Age | Liver | Diagnostic grading | T | N | M | TNM | Stage |
| --- | --- | --- | --- | --- | --- | --- | --- | --- | --- |
| 1 | M | 65 | Liver | Hepatocellular carcinoma | T2 | N0 | M0 | T2N0M0 | II |
| 2 | M | 47 | Liver | Hepatocellular carcinoma | T2 | N0 | M0 | T2N0M0 | II |
| 3 | M | 68 | Liver | Hepatocellular carcinoma | T1 | N0 | M0 | T1N0M0 | I |
| 4 | M | 52 | Liver | Hepatocellular carcinoma | T3 | NX | M0 | T3NXM0 | IIIA |
