# Supplementary material for: METTL3 and STAT3 form a positive feedback loop to promote cell metastasis in hepatocellular carcinoma
Source: Cell Commun Signal. 2023 May 25;21:121. doi: 10.1186/s12964-023-01148-7 (PMC10210303; doi:10.1186/s12964-023-01148-7)
Supplement: Supplementary file 7 — Additional file 6. [file 12964_2023_1148_MOESM6_ESM.docx]

**Supplementary Information (SI) for:**

**METTL3 and STAT3 form a positive feedback loop to promote cell metastasis in hepatocellular carcinoma**

**Materials and methods**

**Plasmid construction and small interference RNA (siRNA)**

The 3'UTR of STAT3 mRNA was amplified by PCR using specific primers and inserted into the pGL3-Control vector to generate the pGL3-Control-STAT3-3'UTR (WT), mutation of m6A site (MUT, A was replaced by T), and deletion of m6A site (DEL) constructs. The complete human METTL3 cDNA sequence (NM_019852) was separately subcloned into the pEGFP-C2 and pCMV-tag2B vector to generate constructs of GFP-METTL3 and pCMV-METTL3. The catalytic mutant of METTL3 (aa395-398, DPPW/APPA) was cloned based on the pCMV-METTL3. The complete human WTAP cDNA sequence (NM_004906) was subcloned into the pEnter vector to generate the pEnter-WTAP constructs. Then, the fragments of WTAP promoter were amplified by PCR using specific primers and inserted into the pGL3-Basic vector to generate the pGL3-Basic-WTAP promoter (pGL-P1~ pGL-P5). The deletions of STAT3 binding sites (Delete-3~Delete-5) were constructed based on pGL-P4. The related cloning and mutagenesis primers and siRNA sequences were listed in supplementary table S4. All siRNAs used in this study were obtained from RiboBio Co., LTD (China).

**RNA isolation and quantitative reverse-transcription polymerase chain reaction (qRT-PCR)**

RNA was extracted from cells using Trizol reagent (Invitrogen, USA) according to the manufacturer's instructions, and the material and reagents used for the extraction of cellular RNA were RNase-free. RNA concentration was measured using NanoDrop 2000 (Thermo, USA). RNA was reversely transcribed to cDNA using an RNA reverse transcription kit (TaKaRa, Japan). Quantitative real-time PCR analysis was then performed on QuantStudio^TM^ 3 (Applied Biosystems, USA). GAPDH was used as an internal control for the samples. Primers used in qRT-PCR are listed in supplementary table S4.

**Chromatin immunoprecipitation (ChIP)**

ChIP was performed with the Simple ChIP® Plus Sonication Chromatin IP Kit (Cell Signaling Technology) according to the manufacturer's protocol. The cells were crosslinked after treatment with 1% formaldehyde and lysed in a ChIP sonication cell lysis buffer. After centrifugation, the cell nuclear lysate was obtained by treatment with ChIP sonication nuclear lysis buffer and sonication with a Branson 450 ultrasonicator. The sonicated cell lysate was separately incubated with the indicated antibody (anti-STAT3, anti-Histone, or negative control IgG) overnight at 4°C with rotation. The precipitated DNA-protein complexes were collected with ChIP-grade Protein G Agarose. The crosslinking of the immunoprecipitates was reversed at 65°C for 30 min, followed by treatment with RNase A and Proteinase K (TaKaRa, Japan) at 65°C overnight to recover DNA fragments. The extracted DNA samples were purified according to the manufacturer's protocol (Tiangen Biotech, China) and finally subjected to PCR analysis. The antibodies used in the ChIP assay included anti-STAT3 (#8768, Cell Signaling Technology) and anti-Histone3 (#AF0009, Beyotime). The related primers are listed in supplementary table S4.

**Dual luciferase reporter analysis**

Dual luciferase reporter analysis was performed according to the manufacturer’s instructions (Promega). To determine whether METTL3 modulated m6A of STAT3 via the sequenced m6A site, the wild-type (WT) of STAT3 3'UTR, mutant (MUT) and delete (DEL) of the indicated m6A site of STAT3 3'UTR both were amplified by PCR and then cloned into pGL3-Control vector. To elucidate the regulation site in WTAP promoter, the wild-type (WT) of WTAP promoter fragments (pGL-P1~ pGL-P5) were amplified by PCR and then cloned into the pGL3-Basic vector. The deletion (Delete-3~Delete-5) fragments were constructed by deleting the indicated STAT3 binding sites of WTAP promoter. HEK293T cells were inoculated in 24-well plates and transfected with the indicated plasmids or siRNAs using Liposome 2000 (Invitrogen). The Renilla luciferase (pRL-TK) plasmid is cotransfected into cells as an internal control. Cells were harvested after transfection for 48h and the activity analysis of dual luciferase in indicated groups was performed according to the manufacturer’s instructions (Promega).

**RNA immunoprecipitation (RIP)**

The cells were washed 3 times with ice-cold PBS, then collected by centrifugation and re-suspended in freshly prepared RIP lysis buffer. After incubating on ice for 5 min, the cell lysates were collected in RNase-free EP tubes and could be stored at -80°C before being centrifuged. The lysate was then rapidly lysed at 37°C and centrifuged at 20,000 g for 10 min at 4°C to obtain the supernatant lysate. The 5 μg antibodies (or negative control IgG) accompanied with 75 μl protein A+G beads (Invitrogen) were added to the lysates and then incubated overnight at 4°C. On the second day, the agarose was washed by RIP washing buffer for 6-8 times and added with Proteinase K. Then, the immunoprecipitated RNA was extracted with Phenol: chloroform purification, and analyzed by qRT-PCR. The antibodies used in RIP assay including anti-METTL3 (#ab195352, Abcam), anti-RPL10A (#16681-1-AP, proteintech) and anti-eIF3b (#10319-1-AP, proteintech). The related primers are listed in supplementary table S4.

**Subcellular fractionation assay**

The treated cells were collected and resuspended with 1 ml PBS. The cell suspension was divided into whole cell lysate (WCL, 200 μl) and nucleus (Nuc, 800 μl) groups. The WCL group cells were treated with RIPA lysis buffer (PMSF added at 1:100) for 5 min and centrifuged at 14000 rpm for 30 min. The supernatant was collected in a new EP tube as a WCL sample. The cells in the Nuc group were collected and gently resuspended with separation buffer (10 mM HEPES, 1.5 mM MgCl2, 10 mM KCl, 0.5 mM DTT, 0.5% NP-40, pH adjusted to 7.9, PMSF added at 1:100) and placed on ice for 20 min. The cells were centrifuged at 4°C for 5 min at 500 g. After centrifugation, the supernatant was transferred into a new EP tube as a cytoplasmic fraction sample (Cyto). Next, the precipitate was washed twice with separation buffer, centrifuged at 500 g for 5 min at 4°C, then lysed on ice for 5 min with lysis buffer (25 mM Tris, 30 mM NaCl, 1% Triton-X100, pH adjusted to 7.5, PMSF added at 1:100) as the nuclear fraction sample for the Nuc group.

**Immunofluorescence (IF) staining**

The treated cells were fixed with ice-cold 4% paraformaldehyde for 10 min, and washed with PBS for 3 times. Then the cells were treated with 0.5% TritonX-100 for 15 min. After washing with PBS, the cells were blocked in 3% BSA for 1 h. Subsequently, cells were incubated with the anti-METTL3 (#67733-1-Ig, proteintech, China) and anti-STAT3 (#8768, Cell Signaling Technology, USA) antibodies at 4°C overnight, and then incubated with the secondary antibodies iFluor^TM^ 488 Conjugated goat anti-mouse IgG antibody (#HA1125, HuaBio, China) and Alexa Fluor 647 Conjugated goat anti-rabbit IgG antibody (#HA1106, HuaBio) for 1 h at room temperature. The incubation process of secondary antibody needed to avoid light. After incubation, cells were washed with PBS and incubated with DAPI for 5 min. The slides were sealed and then photographed using laser scanning confocal microscopy (Leica, Germany).

**Western blotting analysis**

Western blotting analysis was performed with the standard protocol. Cells were washed twice with 1 ml PBS and lysed on ice for 10 min using RIPA cell lysis buffer containing a mixture of protease inhibitor. Equal amounts of total protein were loaded for Western blotting analysis. Protein extracts were separated by SDS-PAGE gels and transferred to PVDF membranes. The membranes were then subjected to rapid blocking solution (NCM Biotech, China) for 15-30 minutes. Incubate sequentially with primary and secondary antibodies. Antibody binding was detected using chemiluminescence (Millipore). The primary antibodies used in this study included rabbit anti-human STAT3α monoclonal antibody (#8768, Cell Signaling Technology), rabbit anti-human METTL3 monoclonal antibody (#ab195352, Abcam, UK), mouse anti-human WTAP monoclonal antibody (#60188-1-Ig, proteintech, USA), rabbit anti-human LaminB1 polyclonal antibody (#12987-1-AP, proteintech, USA), mouse anti-human β-Actin monoclonal antibody (#AF0003, Beyotime, China), rabbit anti-human Tubulin polyclonal antibody (#AF0001, Beyotime, China), anti-GFP (Sigma-Aldrich, USA), anti-human ZC3H13 (DF4623, Affinity, USA) and rabbit anti-human Ki67 monoclonal antibody (#ab16667, Abcam). The Image J software was used to quantify the intensity in Western blot analysis.

**Co-Immunoprecipitation (Co-IP)**

The treated cells were harvested and lysed using lysis buffer (50 mM Tris-HCl pH 7.5, 150 mM NaCl, 1 mM EDTA, 0.5% Triton X-100, 10% Glycerine, 1 mM protease inhibitor PMSF) for 20 min at 4°C with constant shaking. After centrifugation, the supernatant lysate was collected and incubated with 50 µl protein A+G beads (Invitrogen, USA) along with 5 µg of the indicated antibody (or negative control IgG) for 4 h at 4°C. The agarose beads were washed with washing buffer 8-10 times. Then, Elution buffer (0.1M Glycine-HCl, pH 3.0) was added, shaken gently and centrifuged, and the supernatant was collected and neutralized with 1 M Tris-HCl (pH 7.5) containing 1.5 M NaCl. The samples were diluted by adding the appropriate 5×loading buffer and boiled for 10 min at 100°C, then resolved for SDS-PAGE followed by Western blotting analysis.

**Supplementary figure legends**


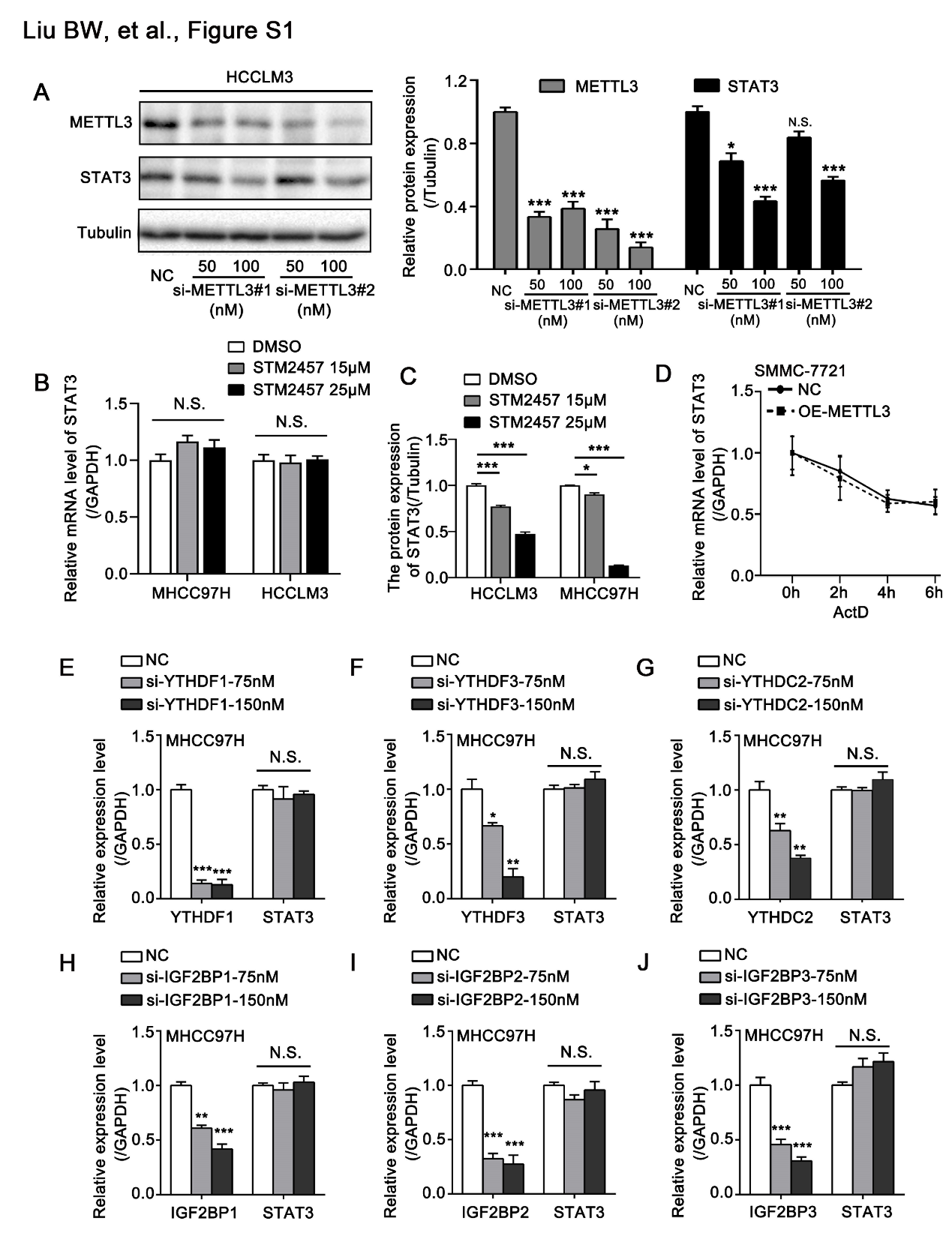


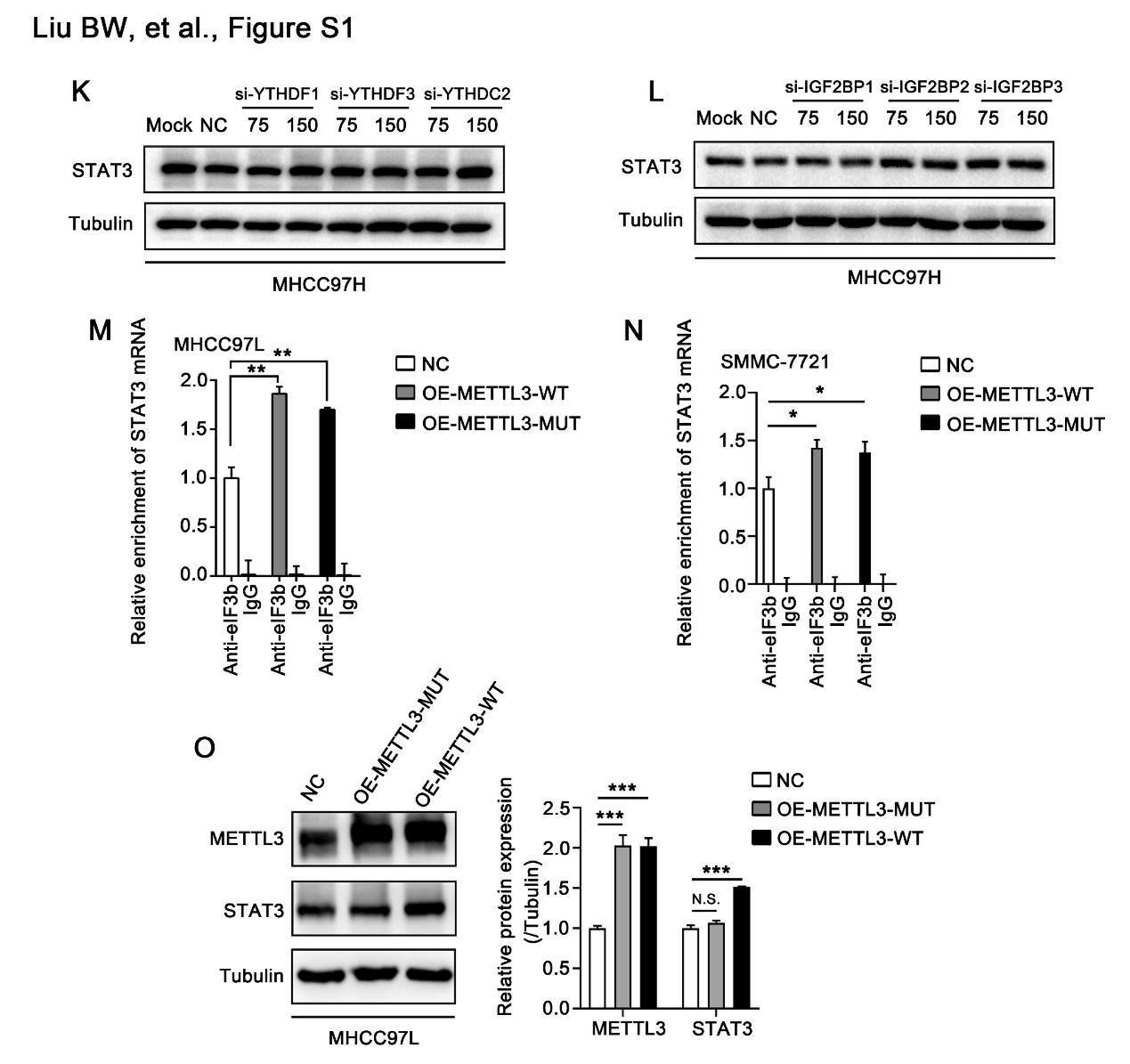


**Fig. S1 METTL3 upregulates STAT3 by promoting the translation of STAT3 mRNA. A** Western blotting analysis of METTL3 and STAT3 in HCCLM3 cells transfected with si-Control or si-METTL3. The right panel shows the quantification of the intensity relative to tubulin. **B** qRT-PCR analysis of STAT3 mRNA level in MHCC97H cells treated with the indicated concentration of STM2457 for 48h. **C** The quantification of the intensity relative to tubulin in Fig. 4G. **D** qRT-PCR analysis of STAT3 mRNA stability in SMMC-7721 cells upon treatment with transcription inhibitor Actinomycin-D (ActD) for the indicated timepoints. The cells were transfected with empty vector or METTL3. **E-J** qRT-PCR analysis of YTHDF1 (E), YTHDF3 (F), YTHDC2 (G), IGF2BP1 (H), IGF2BP2 (I), IGF2BP3 (J) and STAT3 in MHCC97H cells transfected with the indicated siRNAs. **K, L** Western blotting analysis of STAT3 protein level in MHCC97H cells transfected with the indicated siRNAs. The concentration of siRNA is nM. **M, N** qRT-PCR analysis of STAT3 mRNA enrichment in anti-eIF3b immunoprecipitated RNA in MHCC97L (M) and SMMC-7721 (N) cells transfected with the indicated plasmids. **O** Western blotting analysis of STAT3 protein level in MHCC97L cells transfected with the indicated plasmids. The right panel shows the quantification of the intensity relative to tubulin. All experiments were repeated at least three times. Error bars represent mean±SD. **P* < 0.05, ***P* < 0.01, ****P* < 0.001 by 2-tailed Student’s *t-*test.


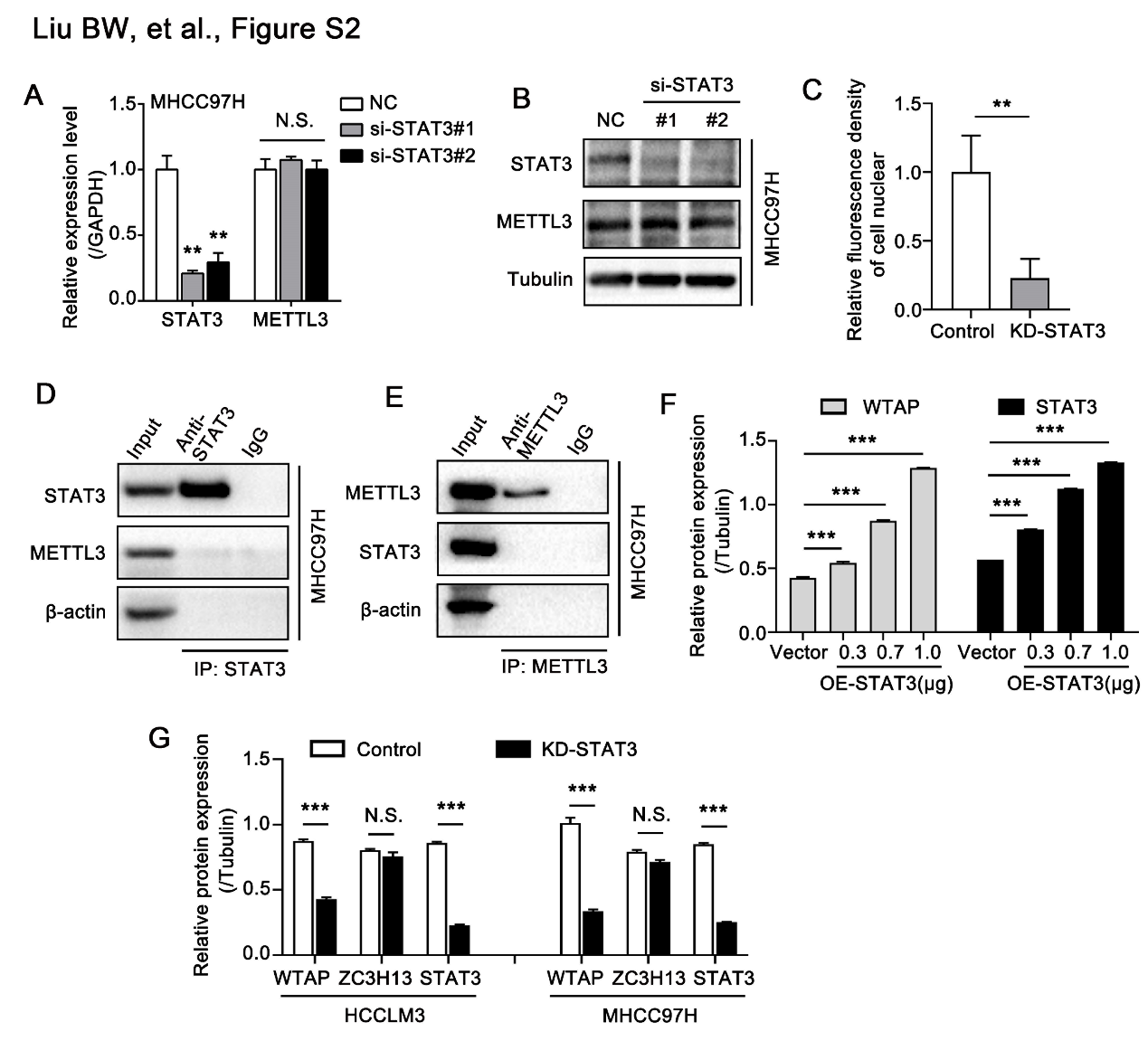


**Fig. S2 STAT3 regulates the nuclear localization of METTL3 *via* WTAP. A, B** qRT-PCR (A) and Western blotting (B) analysis of STAT3 and METTL3 in MHCC97H cells transfected with the indicated siRNAs. **C** The quantification of nuclear fluorescence intensity of anti-METTL3 cell in Fig.5B. **D, E** Co-IP analysis of the interaction between METTL3 and STAT3 in MHCC97H cells. **F** The quantification of the intensity relative to tubulin in Fig. 5E. **G** The quantification of the intensity relative to tubulin in Fig. 5F. All experiments were repeated at least three times. Error bars represent mean±SD. *P < 0.05, **P < 0.01, ***P < 0.001 by 2-tailed Student’s t-test.


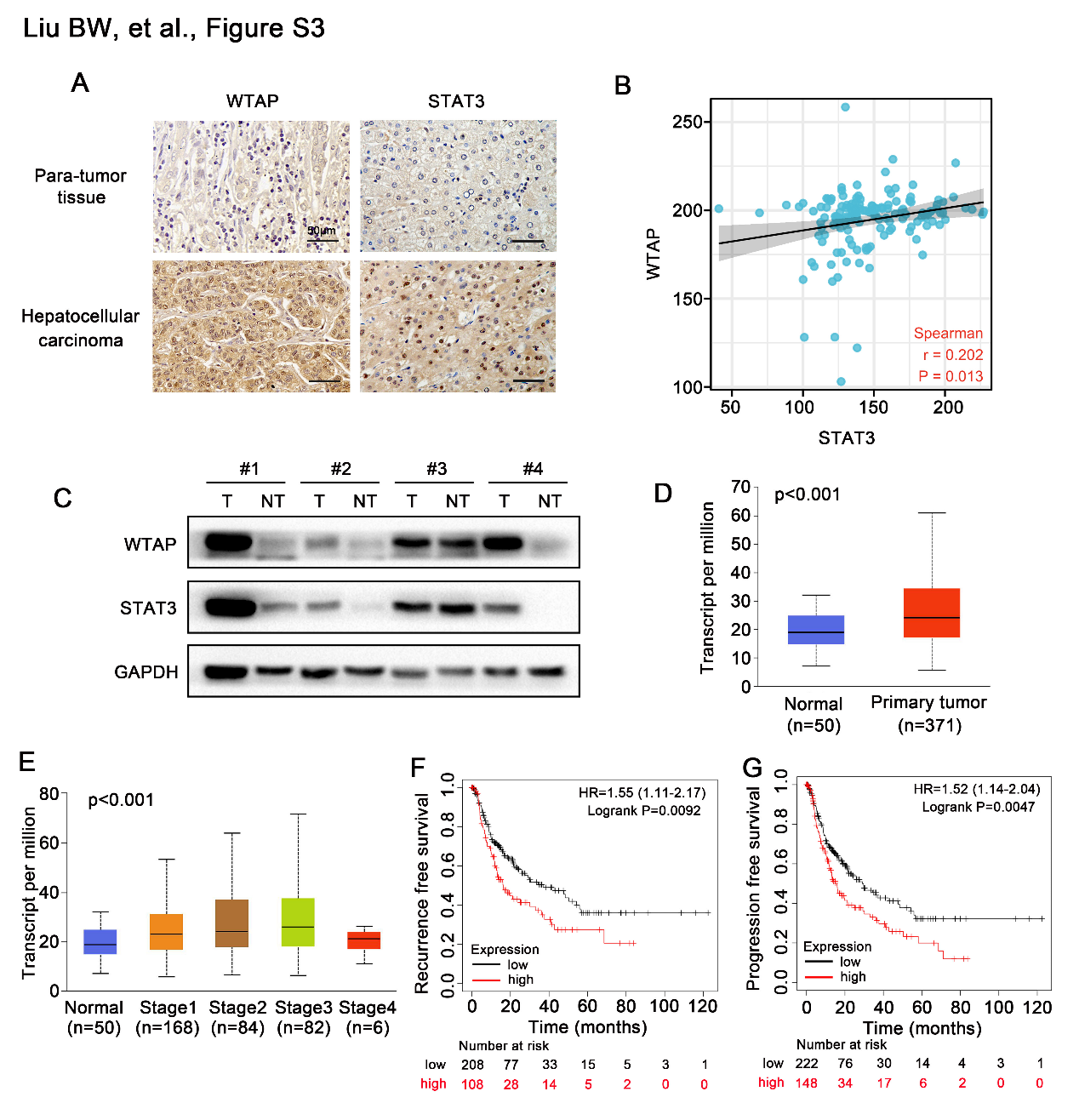


**Fig. S3 WTAP is overexpressed and correlated with STAT3 in HCC tissues. A** IHC staining of WTAP and STAT3 in tissue microarray containing 50 paired liver cancer tissues. Scale bar, 50 μm. **B** The association between the expression levels of STAT3 and WTAP in the tissue microarray was statistically analyzed by Spearman correlation analysis, r=0.202, p<0.05. **C** Western blotting analysis of STAT3 and WTAP in 4 paired HCC tissues (T) and non-tumor tissues (NT). **D** Analysis of WTAP expression in 371 HCC tissues and 50 normal liver tissue samples in the TCGA database, *p*<0.001. **E** Correlation analysis between WTAP expression with clinical stage in HCC patients, *p*<0.001. **F, G** Correlation analysis between WTAP expression with recurrence-free survival (F) and progression-free survival (G) in HCC patients was assessed by the Kaplan-Meier Plotter online tool.


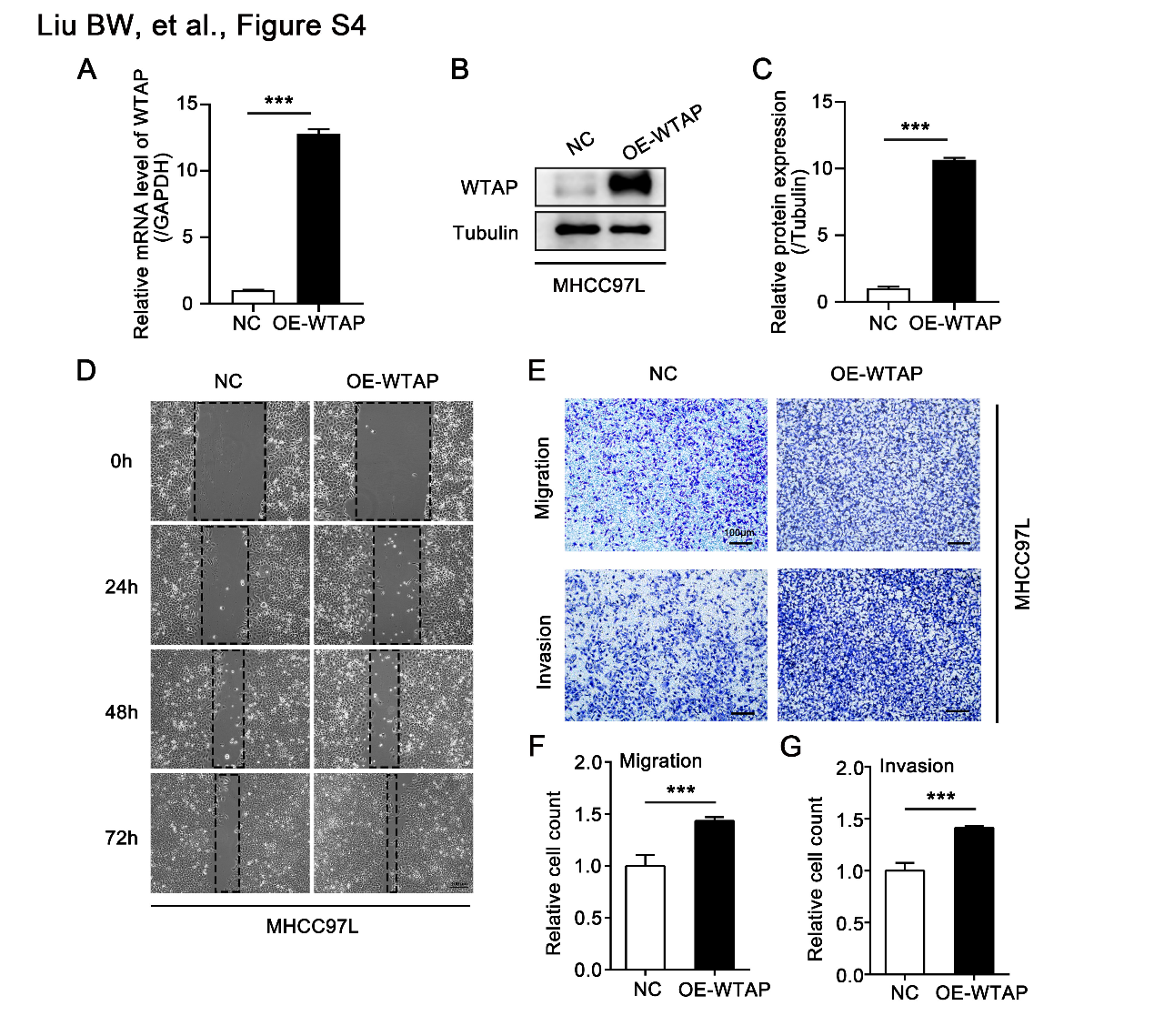


**Fig. S5 WTAP promotes migration and invasion in MHCC97L cells. A** qRT-PCR analysis of WTAP in MHCC97L cells transfected with the indicated plasmids. **B, C** Western blotting analysis (B) of WTAP in MHCC97L cells transfected with the indicated plasmids. (C) shows the quantification of the intensity relative to tubulin. **D** Wound healing assay of the migration ability in MHCC97L cells transfected with indicated plasmids. **E-G** Transwell assay of the migration and invasion abilities in MHCC97L cells transfected with indicated plasmids. Error bars represent mean±SD, **P* < 0.05, ***P* < 0.01, ****P* < 0.001 by 2-tailed Student’s *t-*test.
